# MEGH-DDI: MolT5-driven edge-aware graph learning with Hop2Token representation for drug–drug interaction prediction

**DOI:** 10.1371/journal.pcbi.1014393

**Published:** 2026-10-14

**Authors:** Li Zhou, Yun Zuo, Wenying He, Zhaohong Deng

**Affiliations:** 1 School of Artificial Intelligence and Computer Science, Jiangnan University, Wuxi, China; 2 School of Artificial Intelligence, Hebei University of Technology, Tianjin, China; Wesleyan University, UNITED STATES OF AMERICA

## Abstract

Drug-drug interactions (DDIs) are critical in pharmacology, as synergistic or antagonistic effects between co-administered drugs can enhance therapeutic outcomes or cause toxicity, posing major challenges for drug safety assessment and new drug development. Current molecular representation methods often fail to capture dynamic substructure relationships or semantic interactions among functional groups. To overcome these limitations, this study proposes MEGH-DDI, a multimodal framework that integrates complementary molecular representation learning with graph-structure dynamic perception. The framework employs a dual-channel semantic module that combines Mol2vec-based substructure representations with MolT5 chemical language embeddings, while SHAP-based dynamic feature selection reduces redundancy. For fine-grained structural modeling, we introduce an edge-aware GCNII equipped with bond-level attention to distinguish different types of chemical bonds. Furthermore, the Hop2Token module converts multi-hop neighborhoods into token sequences, enabling Transformer-based modeling of high-order substructure dependencies. Experimental results demonstrate that MEGH-DDI achieves competitive performance across both transductive and inductive settings. On the DrugBank dataset, MEGH-DDI attains an accuracy of 98.36% in the transductive task, outperforming earlier baselines and surpassing recent state-of-the-art methods, with both AUC and AP exceeding 99.7%. On the ChChMiner dataset, MEGH-DDI achieves an F1 score of 99.05%, improving over the best existing method by 2.74 percentage points. In the inductive settings, MEGH-DDI achieves competitive performance across most evaluation metrics on both datasets, showing advantages over competing methods on several key metrics. These results demonstrate the effectiveness of MEGH-DDI in predicting DDI involving previously unseen drugs and its potential to generalize to complex DDI prediction scenarios. To enhance interpretability, MEGH-DDI incorporates a three-level mechanism spanning atoms, chemical bonds, and topological paths, highlighting key atoms and bond contributions. Overall, MEGH-DDI provides an efficient, accurate, and interpretable solution for drug safety assessment and new drug discovery. Code and datasets are available at https://github.com/Lilyjoys/MEGH-DDI, and a free web platform is provided at http://www.edgh-ddi.com.

## Author summary

Drug–drug interactions (DDIs) are critically important in pharmacology, as co-administered drugs can produce synergistic or antagonistic effects that significantly affect therapeutic outcomes and patient safety. However, existing computational methods often struggle to capture the complex semantic and structural dependencies among molecular substructures, particularly in scenarios involving unseen drugs or small-sample datasets. To overcome these limitations, we propose **MEGH-DDI**, a multimodal and interpretable framework for DDI prediction. MEGH-DDI integrates molecular semantic information and graph structural features through a dual-channel design: at the semantic level, it combines Mol2vec substructure embeddings with MolT5 chemical language representations and employs SHAP-based dynamic feature selection to remove redundant features; at the structural level, it introduces an edge-aware GCNII with bond-level attention and leverages the Hop2Token module to serialize multi-hop neighborhoods, enabling Transformer-based modeling of high-order structural dependencies.

Extensive experiments show that MEGH-DDI achieves competitive performance in both transductive and inductive tasks. On the small and sparse ChChMiner dataset, it attains an F1 score of **99.05%** in the transductive task and significantly outperforms existing methods in inductive evaluations. Its three-level interpretability mechanism, spanning atoms, chemical bonds, and topological paths, provides intuitive insights into the model's predictions and facilitates the analysis of molecular features associated with DDI prediction.

## 1 Introduction

The rapid advancement of modern medicine has led to the development of numerous novel drugs, offering new hope for the treatment of many intractable diseases. However, in the face of complex conditions such as cancer and neurodegenerative disorders, clinical practice still encounters significant therapeutic limitations. Traditional monotherapy, due to its single-target nature, not only struggles to address the complex mechanisms underlying these diseases but also tends to induce drug resistance [1]. This has prompted the development of multi-drug combination therapy, which significantly improves treatment precision by synergistically modulating multiple pathological pathways [2]. However, it is noteworthy that the administration of multiple drugs may induce DDIs, potentially leading to either synergistic or antagonistic effects [3]. In other words, the interactions arising from multi-drug administration may either enhance therapeutic efficacy or diminish drug effectiveness due to antagonistic effects. Furthermore, DDIs carry the risk of inducing toxic side effects [4]. Recent studies have warned that tirzepatide may alter the absorption kinetics of co-administered drugs by delaying gastric emptying, and its combination with 6-mercaptopurine has been associated with a potential risk of liver injury. In addition, the study revealed competitive interactions at the level of protein binding. For instance, when co-administered with sodium valproate, competition for plasma protein binding sites may increase the concentration of unbound drugs, thereby triggering toxic reactions. More importantly, the pronounced weight-reducing effect of this drug may influence the metabolism of medications requiring weight-based dose adjustment, such as levothyroxine, thereby posing a risk of overdose toxicity. Statistics indicate that DDIs are associated with approximately 30% of adverse drug reaction (ADR) events [5]. A recent retrospective study on tirzepatide-related adverse drug reactions (ADRs) revealed that among 43 reported cases, 44% involved polypharmacy scenarios, raising a significant concern regarding the safety of combined drug administration [6]. In new drug development, systematic evaluation of drug–drug interactions has become a crucial step to ensure clinical safety. A comprehensive understanding of DDI mechanisms not only helps optimize therapeutic regimens but also provides important scientific evidence for innovative drug development, holding profound significance for improving healthcare quality and ensuring patient safety.

In the field of drug-drug interaction research, traditional approaches mainly rely on time-consuming in vivo and in vitro experiments, which assess potential risks by monitoring changes in plasma drug concentrations and pharmacokinetic parameters. However, these methods suffer from limitations such as insufficient sample representativeness, unavoidable measurement errors, and high costs, making them inadequate to meet the large-scale demands of modern drug development. In recent years, breakthroughs in Artificial Intelligence (AI) technology have paved new avenues for DDI research, with Machine Learning (ML), particularly Deep Learning (DL) techniques, demonstrating significant advantages in prediction tasks. Early studies primarily focused on prediction by constructing drug similarity matrices and employing traditional algorithms such as logistic regression [7]. Subsequently, Cheng et al. [8] developed a network inference framework that integrates multi-source heterogeneous data, combining multidimensional features such as drug phenotypes and genomics, which substantially improved prediction accuracy. However, these methods still have room for improvement due to their reliance on the quality of feature engineering. The emergence of knowledge graph techniques has provided a novel approach to addressing this challenge. By integrating large-scale biomedical knowledge bases such as BioKG [9], researchers are able to construct drug networks that capture rich semantic relationships. A hybrid knowledge graph was constructed based on DrugBank [10] and PharmGKB [11], and a Conv-LSTM framework, combining convolutional neural networks (CNNs) with long short-term memory (LSTM) networks, was proposed for predicting drug–drug interactions. Subsequent studies further optimized knowledge representation by employing graph embedding techniques to map drug nodes into dense vector spaces, thereby significantly enhancing feature representation capabilities [12]. Notably, the SumGNN model [13] achieved breakthrough improvements in both predictive performance and interpretability by incorporating a self-attention mechanism. In particular, for adverse drug reaction prediction, advanced models have reached accuracies exceeding 87% [14], highlighting the substantial potential of artificial intelligence in the field of pharmacology. Currently, deep learning–based DDI prediction research is driving computational pharmacology into a new era. Since drug molecules and their interaction networks can be represented as complex graph structures, graph neural networks (GNNs), which are well-suited for processing irregular graph data, have been widely applied to uncover patterns from DDI data, enabling efficient prediction of DDIs. In 2022, the SSI-DDI model developed by Nyamabo et al. [15] achieved a breakthrough by decomposing overall drug interactions into substructure-level interactions, enabling direct processing of raw molecular graph data. In the same year, Yang et al. [16] proposed the SA-DDI model, which incorporates a dynamic substructure attention mechanism capable of adaptively capturing the topological features of irregular functional groups with high precision.

Feature fusion techniques have also made significant progress: Lin et al. [17] developed the MDF‑SA‑DDI model, which integrates four heterogeneous networks to generate drug feature vectors and employs a self-attention mechanism to achieve deep fusion of multi-source information. Subsequently, Nyamabo et al. [18] developed the GMPNN‑CS model, which employs a gated message passing network to achieve learnable partitioning of chemical substructures. Notably, Li et al. [19] proposed the DSN‑DDI model in 2023, which for the first time established a dual-view learning framework. By iteratively optimizing local and global representations, it systematically addresses the limitations of traditional methods that neglect atom-level interactions. The DDI‑GCN model [20] applied a graph convolutional network architecture to achieve accurate prediction of complex interactions among multiple drugs. By combining molecular graph encoding with an attention mechanism, the model enables efficient fusion and analysis of drug features, providing a novel approach for DDI prediction. To address the critical issue that existing models largely overlook chemical substructure interaction information, Ma and Lei (2023) [21] developed the landmark DGNN‑DDI dual graph neural network model. The model incorporates two major innovative modules: (1) an intelligent substructure recognition system based on SA‑DMPNN, which can dynamically capture key regions of molecules with functional activity; (2) a substructure pair interaction analysis engine, which transforms traditional molecule-level prediction into a fine-grained assessment of substructure–substructure interactions, achieving a dual breakthrough in both predictive accuracy and mechanistic interpretability. The PEB‑DDI framework proposed by Shen et al. [22] represents another significant advancement in the field of DDI prediction. This intelligent system can autonomously switch computation modes based on task characteristics: it employs a TransformerConv-driven multimodal fusion strategy for known drug scenarios, while activating a GAT-based heterogeneous graph learning architecture for predictions involving novel drugs, demonstrating exceptional adaptability across different contexts.

Current approaches still exhibit significant limitations. On one hand, most models primarily rely on 2D molecular representations, failing to adequately capture the hierarchical structure and semantic dependencies within molecules. On the other hand, traditional methods are insufficient in effectively combining atom-level features with neighborhood structures and generally overlook the semantic interactions across different substructures.

To address these challenges, this study proposes an innovative solution named MEGH-DDI. As illustrated in Fig 1, the model architecture comprises two core components: a molecular global semantic module and a graph structural feature extraction module. In the molecular global semantic module, the model integrates Mol2vec and MolT5 features. Mol2vec learns substructure co-occurrence patterns from a large-scale molecular corpus to generate semantic-level vectors that capture structural commonalities and functional group information, while MolT5 treats SMILES sequences as a chemical language and employs a deep Transformer attention mechanism to extract both syntactic and semantic features.

**Fig 1:**
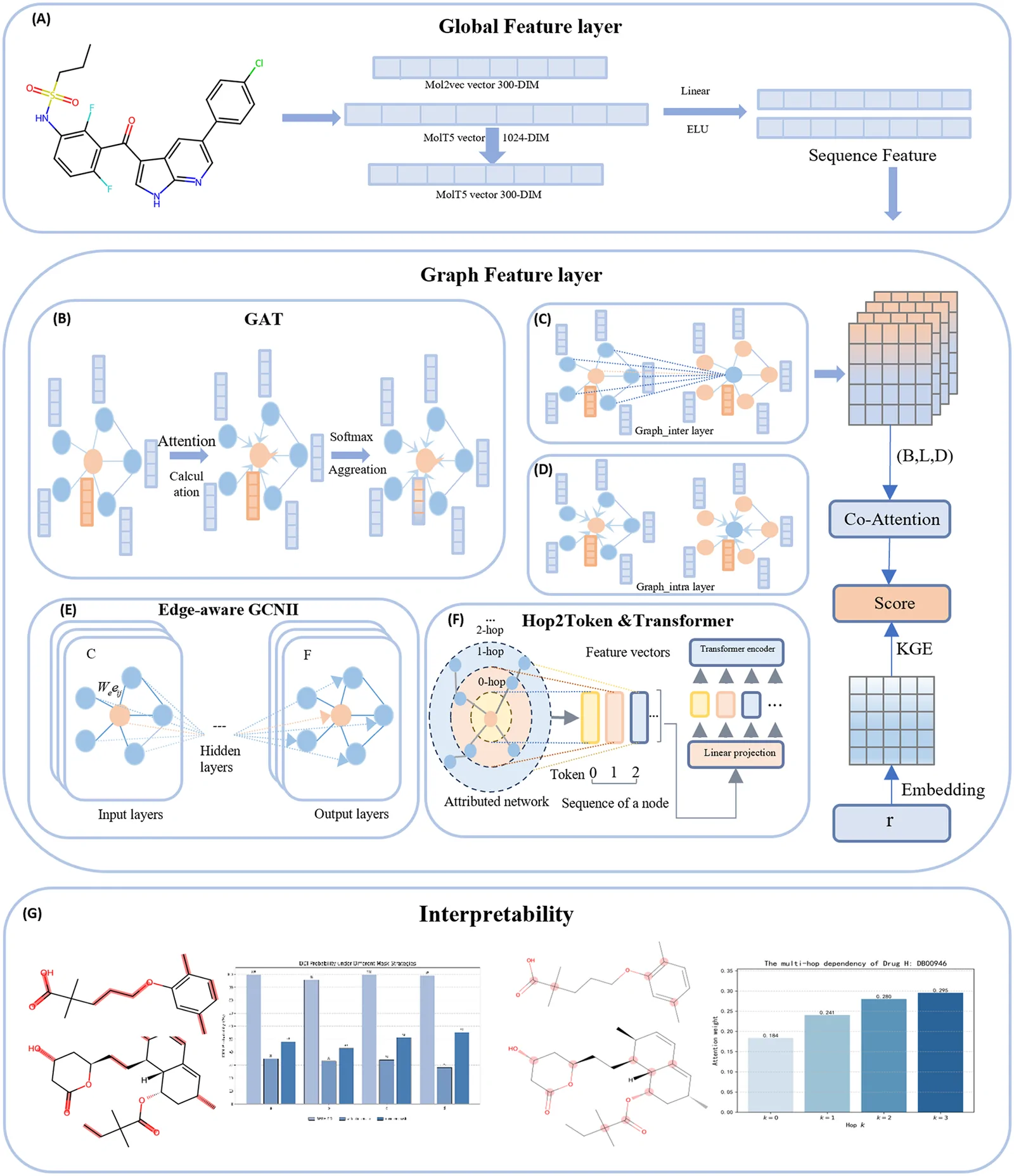
Architecture of the MEGH-DDI model. (A) extracts global molecular features, including substructure-level Mol2vec embeddings and cross-modal pre-trained MolT5 representations. (B-F) extract graph structural features, integrating a GAT with Edge-aware GCNII to enhance chemical bond modeling, while employing Hop2Token and Transformer to capture semantic contributions across different neighborhood ranges. (G) provides interpretability at the atom level, chemical bond level, and multi-hop structural range.

The graph structural encoder consists of two complementary branches. The first branch applies GATConv to aggregate local neighborhood information, and the resulting node representations are subsequently fed into Hop2Token to encode multi-hop neighborhood information. In parallel, an edge-aware GCNII branch performs deep residual graph propagation while incorporating edge-derived weights into the normalized message-passing process. The representations produced by the two branches are then integrated to obtain a comprehensive structural representation. The Hop2Token-derived representations are further processed by a Transformer encoder to capture dependencies across different neighborhood ranges. Next, the Graph-intra Attention and Graph-Inter Attention within the block are employed to respectively enhance the structural consistency within individual drug molecules and the interaction features between drug pairs. The graph pooling and readout layers capture multi-level graph-level representations, and the prediction task is ultimately completed through interactive fusion and KGE-based scoring. This multi-perspective, multi-level architecture simultaneously captures local chemical structures of drugs, global topological patterns, and cross-drug interaction mechanisms, thereby significantly improving the accuracy of DDI prediction. To enhance model transparency, this study further conducts a systematic interpretability analysis of MEGH-DDI. By employing attention mechanisms and module-level analysis, the model can generate explanatory signals at the atom level, bond level, and multi-hop neighborhood levels. This process verifies its capability to identify drug interaction mechanisms and provides traceable and interpretable evidence to support rational clinical drug use.

## 2 Materials and methods

### 2.1 Dataset and task settings

In this study, the MEGH-DDI model was evaluated on two benchmark DDI datasets, DrugBank and ChChMiner. DrugBank [10] is derived from drug labels provided by the FDA and Health Canada, where different types of interactions describe how one drug influences the metabolism of another. The dataset contains 1,706 drugs and 191,808 interactions covering 86 types of interactions. Since DrugBank includes only positive samples, this study follows Shen et al. [22] by generating an equal number of negative samples based on a Bernoulli sampling distribution to maintain balance. The ChChMiner dataset [23] contains 997 drugs and 33,669 interactions, including 21,486 positive samples and 12,183 negative samples. Among them, 798 are known drugs and 199 are unknown drugs, with the interaction types categorized into two classes.

Both transductive and inductive evaluations were conducted on the DrugBank and ChChMiner datasets. The detailed data splits are presented in Tables 1 and 2, respectively, with fold 0 used as an example for the inductive setting of ChChMiner. In the transductive setting, DrugBank was evaluated using three independent data splits (folds 0–2) generated with different random seeds. For each split, the predefined training and test sets were used, and the training set was further divided into training and validation subsets at an 8:2 ratio using stratified random sampling. For ChChMiner, the dataset was divided into training, validation, and test sets at a ratio of 3:1:1, while maintaining the original proportions of positive and negative samples. In the inductive setting, three independent splits (folds 0–2) were also used. For each fold, drugs were divided into 80% known drugs and 20% new drugs. The training set consisted only of interactions involving known drugs, whereas the test set was divided into S1, representing interactions between two new drugs, and S2, representing interactions between a new drug and a known drug. The model was trained and evaluated independently on each fold, and the final results were reported as the average across the three folds.

**Table 1:** DrugBank dataset splits.

Task type	Split	Positive	Negative
Transductive/Inductive	Total	191808	191808
	Train	122757	122757
	Val	30689	30689
	Test	38362	38362

**Table 2:** ChChMiner dataset splits (The inductive split is shown using fold 0 as an example).

Task Type	Split	Positive	Negative	Task Type	Split	Positive	Negative
Transductive	Total	21486	12183	Inductive	Total	21486	12183
	Train	12784	10651		Train	13379	7901
	Val	4178	943		Test-S1	946	466
	Test	4524	589		Test-S2	7161	3816

### 2.2 Problem definition

In this study, the DDI prediction problem is formulated as a binary classification task. For a given drug pair $\left(d_{i},d_{j}\right)$, where $d_{i}$ and $d_{j}$ represent two different drugs, a probabilistic discriminative model is constructed to predict whether an interaction exists between the drug pair. The corresponding binary label is defined as y∈{0,1}, where y=1 indicates that the drug pair has a known DDI, while y=0 indicates that the drug pair is considered non-interacting. The model computes a scalar interaction score $s_{ij}$, representing the likelihood that the drug pair exhibits a DDI. When the threshold is set to τ=0.5, the interaction is predicted to exist (label = 1) if $s_{ij}\geq \tau$, and to be absent (label = 0) otherwise.

Although the DrugBank dataset contains 86 distinct interaction types, these types are not treated as separate prediction classes in this study. Instead, they represent the diversity of known DDI relationships in the original dataset. For the binary prediction task, all observed drug pairs with any of the 86 interaction types are assigned the positive label y=1, whereas the drug pairs generated through negative sampling are assigned the negative label y=0. Therefore, the model focuses on predicting the existence of a DDI rather than identifying the specific interaction type.

To comprehensively analyze the chemical properties and interaction patterns of drugs, this study designs a representation framework that integrates multi-dimensional features. The framework combines global molecular descriptors, semantic features, and graph structural information to fully capture both the structural and semantic characteristics of drugs, providing a more complete knowledge basis for DDI prediction. The RDKit [24] toolkit was used to convert SMILES strings into molecular graph structures. A molecular graph consists of nodes and edges, denoted G=(V,E), where the node set V represents the atoms in the molecule and the edge set E represents the chemical bonds. Node and edge features are derived from relevant chemical knowledge [22], with each atom $v_{i}\in V$ represented by a feature vector $x_{i}\in R^{\text{1}\times \text{5}\text{5}}$ and each chemical bond $\left(v_{i},v_{j}\right)\in E$ represented by a feature vector $e_{ij}\in R^{\text{1}\times \text{1}\text{7}}$. Initial node and edge embeddings are obtained through linear transformations followed by nonlinear activation: $h_{i}^{\left(\text{0}\right)}=\sigma \left(W_{h}x_{i}\right)$, $e_{ij}^{\left(\text{0}\right)}=\sigma \left(W_{e}e_{ij}\right)$, where$W_{h},W_{e}$ are learnable parameters and σ(·) denotes the activation function ReLU.

In addition, this study innovatively integrates two unsupervised molecular representation techniques: Mol2vec and MolT5. Mol2vec, inspired by word embedding methods in natural language processing, treats molecular substructures as “words” and generates a global vector representation for the entire molecule that reflects both its functional and structural similarities. In contrast, MolT5 leverages the deep attention mechanisms of the Transformer architecture to extract richer chemical semantic features from SMILES strings, producing molecular embeddings with stronger representational capacity. In the experimental design, Mol2vec features and MolT5 features are applied to both transductive prediction and inductive reasoning scenarios. To capture the microscopic mechanisms of DDIs more effectively, we constructed a graph-interaction view, namely a drug-drug bipartite graph. For a given DDI triplet $\left(d_{x},\;r,\;d_{y}\right)$, the node set of this bipartite graph consists of all atoms from both drugs $d_{x}$ and $d_{y}$. Edges in the graph connect atoms from $d_{x}$ to atoms from $d_{y}$, representing candidate atom-pair connections used for cross-graph attention. This interaction graph, together with the graph-intra structure of each individual drug, forms the basis for a dual-view learning framework for DDI prediction [19].

### 2.3 Feature extraction

#### 2.3.1 Mol2vec.

This study introduces an unsupervised molecular representation framework, Mol2vec [25], which overcomes the limitations of traditional molecular fingerprints and enables deep and intelligent characterization of drug molecular structural features. Mol2vec innovatively adapts the core paradigm of Word2Vec [26] from natural language processing, decomposing molecular structures into chemically meaningful “substructure vocabularies”. Through a context-based learning mechanism, it obtains distributed vectors for each substructure, thereby capturing co-occurrence patterns and forming continuous molecule-level vector representations. The design of Mol2vec embodies “chemical intuition”, meaning that substructures with similar scaffolds or functional groups are positioned close to each other in the embedding space, thereby reflecting their similarity in chemical functionality. Compared with traditional molecular fingerprints, such as Morgan fingerprints, which suffer from sparsity and bit collision issues, Mol2vec representations are denser and learnable, demonstrating superior performance in various compound property prediction and bioactivity modeling tasks. The implementation of this method comprises three modules, as illustrated in Fig 2.

**Fig 2:**
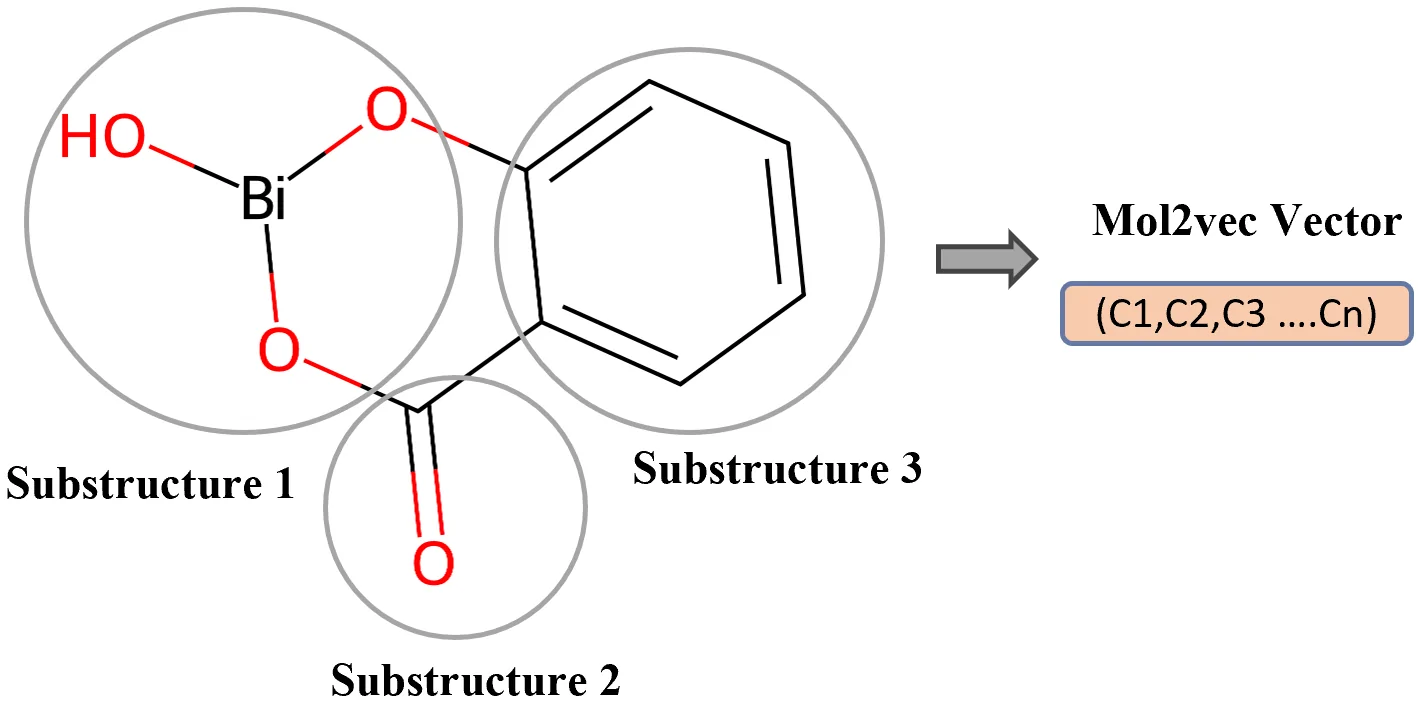
Mol2vec encoding of drugs.

(1) **Generation of Substructure “Sentences”:** First, each molecule is decomposed into multiple substructures using the Morgan fingerprint algorithm, which is based on a radius extension strategy for atomic environments. Each resulting substructure (e.g., an atomic neighborhood with a radius of 1 or 2) is then encoded as a unique identifier (such as C-1, O-3, etc.). Consequently, each molecule is ultimately represented as a “sentence” composed of a sequence of these substructures.
(2) **Training of Substructure Vectors:** Using Word2Vec mechanisms such as Skip-gram or CBOW, distributed vector representations (typically 300 dimensions) of substructures are trained on large-scale molecular corpora (e.g., ChEMBL). The model captures underlying structural co-occurrence patterns and semantic information by maximizing the contextual relationships between substructures.
(3) **Molecular Vector Construction:** After obtaining vector representations for all substructures, the vectors of the substructures of a given molecule are summed or averaged to generate its overall molecular vector. This molecule-level representation preserves the contextual information of the substructures, enabling a more accurate reflection of the local structural features of the drug and the co-occurrence patterns of functional groups.

#### 2.3.2 MolT5.

To more comprehensively capture both structural and semantic information of drug molecules, this study introduces the MolT5 [27] model as a molecular-level feature extraction tool. MolT5 is a cross-modal pre-trained model built on the T5 (Text-to-Text Transfer Transformer) architecture, originally designed to enable bidirectional “translation” between natural language and molecular structures, including molecule description generation and text-based de novo molecular design. The core idea is to treat SMILES molecular structures as a “language” and use self-supervised training to establish deep connections between chemical structures and natural language semantics. MolT5 is pre-trained on large-scale unlabeled data, which not only effectively mitigates the scarcity of high-quality annotated data in the chemical domain but also endows the model with strong structural awareness and language understanding capabilities. For this study, the MolT5 encoder can map input SMILES strings into high-dimensional vector representations that capture both molecular syntax and functional information, thereby providing strong semantic support for downstream drug property prediction tasks. The technical implementation of drug molecular feature extraction is as follows: a pre-trained model released on the HuggingFace platform is used, retaining only its encoder for feature extraction. Specifically, the SMILES representation of the target drug is first input into the MolT5-specific tokenizer, which decomposes it into a sequence of tokens recognizable by the model. Next, the token ID sequence is fed into the encoder to obtain a high-dimensional representation tensor for each token with shape ([batch_size,seq_len,hidden_dim]). This vector can then be used for downstream tasks such as drug activity prediction, structural similarity computation, or clustering analysis.

### 2.4 Feature selection and dimensionality reduction based on SHAP

Although the high-dimensional molecular representations generated by the MolT5 encoder (1024 dimensions in this study) contain rich information, they inevitably introduce computational redundancy and potential noise, which may negatively influence the generalization performance of the model. To enhance the efficiency and stability of downstream prediction tasks while preserving the completeness of molecular representations, this study applies a feature selection strategy to intelligently compress high-dimensional vectors. Unlike traditional unsupervised dimensionality reduction techniques, such as PCA, which rely solely on data distribution, we employ the SHAP (SHapley Additive Explanations) framework to perform supervised feature selection, combining mathematical rigor with task-oriented guidance.

Importantly, the SHAP-based feature selection procedure does not introduce data leakage. The LightGBM model is trained exclusively on the training set, and SHAP values are computed only using training samples. Feature importance is determined based on the mean absolute SHAP values from the training data, and the resulting feature subset is fixed and directly applied to the validation and test sets without recomputation. At no stage are validation or test samples involved in feature selection, thereby ensuring a strictly leakage-free experimental protocol.

The specific implementation procedure consists of two main steps: First, a drug interaction prediction model is constructed using a LightGBM classifier. Subsequently, the SHAP explainer is applied to quantify the contribution weight of each feature to the prediction results. By computing the mean absolute value of the SHAP values for each feature and ranking them, we selected the top 300 key features from the initial 1024-dimensional set. This allowed us to construct a streamlined and effective drug representation vector. This process achieves not only dimensionality reduction from 1024 to 300 features but also ensures that the retained features are the most relevant for the drug interaction prediction task, thereby providing a more efficient and interpretable input space for subsequent modeling.

The experimental evaluation (as shown in Table 3) systematically compares different dimensionality reduction methods for MolT5 features in the transductive DDI prediction task on the ChChMiner dataset, while keeping the inclusion of Mol2vec features and the overall model architecture unchanged. The original 1024-dimensional MolT5 representation achieved an accuracy (ACC) of 98.42%, an area under the ROC curve (AUC) of 99.67%, and an F1-score of 99.04%, providing a strong baseline for comparison. PCA and UMAP achieved higher AUC values of 99.78% and 99.85%, respectively, but showed lower ACC and F1-scores than the baseline, indicating that improved ranking performance did not necessarily translate into better classification performance. ANOVA achieved the highest AUC of 99.91% and maintained a specificity (SP) of 99.87%, while achieving an ACC of 98.89% and an F1-score of 99.37%. Among all evaluated methods, SHAP achieved the highest ACC, F1-score, and sensitivity (SN), reaching 99.11%, 99.49%, and 99.03%, respectively. Compared with the original MolT5 representation, SHAP improved ACC by 0.69 percentage points, F1-score by 0.39 percentage points, and SN by 0.80 percentage points, while increasing AUC to 99.88%. Although its AUC was slightly lower than that of ANOVA and its SP decreased from 99.87% to 99.73%, SHAP demonstrated the strongest overall performance in terms of ACC, F1-score, and SN among the evaluated methods. Furthermore, reducing the MolT5 feature dimensionality from 1024 to 300 provides a more compact representation for downstream prediction. These results support the effectiveness of SHAP-based feature selection in improving selected predictive performance metrics while reducing the dimensionality of molecular representations.

**Table 3:** Experimental results of MolT5 features using various dimensionality reduction methods.

MolT5 (1024–300)	ACC (%)	AUC (%)	F1 (%)	SP (%)	SN (%)
Base	98.42	99.67	99.10	**99.87**	98.23
PCA	97.70	99.78	98.68	98.67	97.57
UMAP	98.06	99.85	98.89	99.84	97.83
ANOVA	98.89	**99.91**	99.37	**99.87**	98.76
**SHAP**	**99.11**	99.88	**99.49**	99.73	**99.03**

## 3 Overview of the model

To comprehensively represent the multi-source features of drug molecules across chemical structures, topological relationships, and inter-graph interactions, this study proposes a multi-level graph learning framework that integrates graph neural network modeling with sequential Transformer-based inference. The framework introduces three key innovations designed to overcome limitations in existing DDI prediction methods: (1) an Edge-aware GCNII module that incorporates bond-type attention into deep graph convolutions to prevent over-smoothing while preserving chemical semantics; (2) a Hop2Token scheme that serializes multi-hop neighborhoods into token sequences, enabling Transformers to capture long-range dependencies across different topological scales; and (3) a multi-modal fusion strategy that combines graph-derived representations with global molecular embeddings (Mol2vec, MolT5) for holistic drug encoding. Through a modular design, the model progressively abstracts drug graphs from local atom-bond structures to global graph-level representations, ultimately enabling inter-drug interaction reasoning. The overall architecture consists of the following key components: first, a graph attention network (GAT) is employed to capture adaptive interaction weights between atoms via self-attention on node features, thereby enhancing the initial feature representations through weighted neighborhood aggregation. Subsequently, the Edge-aware GCNII convolution module balances topological smoothing and feature diversity during multi-layer propagation, with a specific emphasis on enhancing modeling capability for chemical bond information. To overcome the limitations of GNNs in modeling higher-order neighborhoods, Hop2Token and Transformer serialize multi-hop structural information into tokens, enabling explicit learning of the semantic contributions of different neighborhood ranges. At the graph-level stage, intra-graph and inter-graph attention mechanisms establish cross-feature interactions, and SAGPooling selects key substructures to form compact representations. Ultimately, multi-block graph representations are synergistically integrated with knowledge graph embeddings (RESCAL) to enable DDI prediction with model-level attribution.

### 3.1 GAT

GAT represents a breakthrough architecture in the field of graph deep learning, which innovatively integrates the attention mechanism with graph structural modeling. The model captures the varying influence of each node on its adjacent nodes by assigning different weights to neighboring nodes, thereby enabling precise representation and learning of graph-structured data. In GAT, the feature representation of each node is computed by aggregating the features of its neighboring nodes weighted by their corresponding attention scores. This mechanism enables the model to capture complex dependencies within the graph structure and effectively handle the heterogeneity and dynamics of graph data.

First, GAT is employed to update node features. Unlike standard GCNs that assign uniform or static weights to neighboring nodes, GAT dynamically computes attention weights for each neighbor through a self-attention mechanism. Let $h_{i}$ and $h_{j}$ denote the feature representations of nodes i and j, and let $W_{h}$ be the weight matrix for the linear transformation of node features. The attention coefficient between nodes iandjis computed as shown in Equation (1):


$$\begin{array}{c} e_{ij}=LeakyRelu\left(a^{T}\left[W_{h}|h_{i}|W_{h}h_{j}\right]\right) \end{array}$$ (1)

Here, a denotes a learnable attention vector,|| represents the concatenation operation, LeakyRelu is the activation function. The attention coefficients are normalized using the Softmax function to obtain the final attention weights $\alpha_{ij}$, as shown in Equation (2):


$$\begin{array}{c} \alpha_{ij}=\frac{exp\left(e_{ij}\right)}{\sum_{k\in N(i)}exp\left(e_{ik}\right)} \end{array}$$ (2)

Here, $N_{\left(i\right)}$ denotes the set of neighboring nodes of node i, Based on the computed attention weights, the features of neighboring nodes are aggregated to obtain the updated representation of node i, as shown in Equation (3):


$$\begin{array}{c} h_{i}^{\prime }=\sigma \left(\sum_{j\in N(i)}\alpha_{ij}W_{h}h_{j}\right) \end{array}$$ (3)

σ(·) denotes the nonlinear activation function ReLU and $h_{i}^{\prime }$ represents the updated embedding of node i at the current layer.

### 3.2 GCN

The Graph Convolutional Network (GCN) has become a key technique in drug discovery tasks due to its exceptional capability in processing graph-structured data. Through its innovative graph convolution operation, the architecture effectively integrates topological relationships between nodes with their feature information, enabling multi-level semantic representation learning. Specifically, in the DDI prediction task, GCN first constructs a molecular graph based on atomic features and then performs intelligent aggregation of neighborhood features through the normalized adjacency matrix and a learnable weight matrix. The aggregated node embeddings are subsequently transformed using a nonlinear activation function to enhance the representational capacity of the model. Through this hierarchical and progressive information propagation mechanism, GCN gradually integrates local structural features with global topological information, ultimately producing node representations rich in semantic content. Its core computational process can be precisely described by the following mathematical expression (4):


$$\begin{array}{c} H^{\left(l+\text{1}\right)}=\sigma \left({\tilde{D}}^{-\frac{\text{1}}{\text{2}}}\tilde{A}{\tilde{D}}^{-\frac{\text{1}}{\text{2}}}H^{\left(l\right)}W^{\left(l\right)}\right) \end{array}$$ (4)

$\tilde{A}=A+I_{N}$ represents the adjacency matrix A with added self-loops, $I_{N}$ denotes the identity matrix, ${\tilde{D}}^{-\frac{\text{1}}{\text{2}}}$ is the inverse square root of the degree matrix used for normalization. This normalization ensures that each neighbor contributes proportionally during feature aggregation, preventing high-degree nodes from disproportionately influencing the aggregated representation. $W^{\left(l\right)}$ is the weight matrix at the l−th layer for linear transformation of node features, and 𝜎 is the nonlinear activation function ReLU, which introduces nonlinearity to enable the model to capture complex patterns.

### 3.3 Edge-aware GCNII

Notably, as network depth increases, GCNs encounter the challenge of representational homogenization — specifically, the over-smoothing phenomenon. This leads to a gradual loss of discriminative power in node features, thereby significantly constraining the potential for developing deeper models. To address this critical issue, the GCNII architecture [28] innovatively integrates a dual stabilization mechanism. Its initial residual connection technique preserves the original input features, thereby effectively maintaining feature diversity across network layers. Simultaneously, the introduced identity mapping operation significantly enhances the feasibility of training deep networks by stabilizing matrix transformations. Based on these two enhancements, the l−th layer of GCNII can be formally expressed as shown in Equation (5):


$$\begin{array}{c} H^{\left(l+\text{1}\right)}=\sigma \left(\left(\left(\text{1}-\alpha_{l}\right)\tilde{P}H^{\left(l\right)}+\alpha_{l}H^{\left(\text{0}\right)}\right)\left(\left(\text{1}-\beta_{l}\right)I_{n}+\beta_{l}W^{\left(l\right)}\right)\right) \end{array}$$ (5)

As shown in Equation (5), GCNII primarily combines the smoothed representation $\tilde{P}H^{\left(l\right)}$ with the initial residual connection from the input features $H^{\left(\text{0}\right)}$ and incorporates an identity $I_{n}$ mapping into the weight matrix $W^{\left(l\right)}$ of the l−th layer. This design effectively mitigates over-smoothing in deep networks while simultaneously enhancing the stability of parameter updates and overall trainability.

To address the importance of bond features in molecular graph modeling for message passing, this study proposes an Edge-Aware GCNII convolution module, as illustrated in Fig 3. This module preserves the two key mechanisms in GCNII for mitigating over-smoothing, namely the initial residual and identity mapping, while introducing learnable attention weights based on edge features. This allows the neighborhood aggregation process to distinguish between different types of edges. Specifically, the structural and chemical features of each edge in the graph are transformed through a linear mapping to obtain a scalar attention coefficient, which is then incorporated into the standard normalized neighborhood aggregation. The intra-layer coefficients α and θ are then combined to achieve the fusion of inter-layer residuals and initial features. Finally, a nonlinear transformation is applied to generate the node representations for the next layer. The initial node feature matrix is denoted as $H^{\left(\text{0}\right)}\in R^{N\times d}$, where N represents the number of nodes and d denotes the dimensionality of node features. The input node representation of the l−th layer is denoted as $H^{\left(l\right)}\in R^{N\times d}$. Each edge feature is represented as $e_{ij}\in R^{d_{e}}$, where $d_{e}$ is the dimensionality of edge features, and the corresponding edge weight is denoted as $W_{ij}$, the residual coefficient is denoted as α∈[0,1], and the identity mapping coefficient is denoted as θ∈[0,1]. The edge attention score of each edge is computed by linearly mapping the edge feature into a scalar and incorporated into the normalized adjacency matrix, as shown in Equation (6):

**Fig 3:**
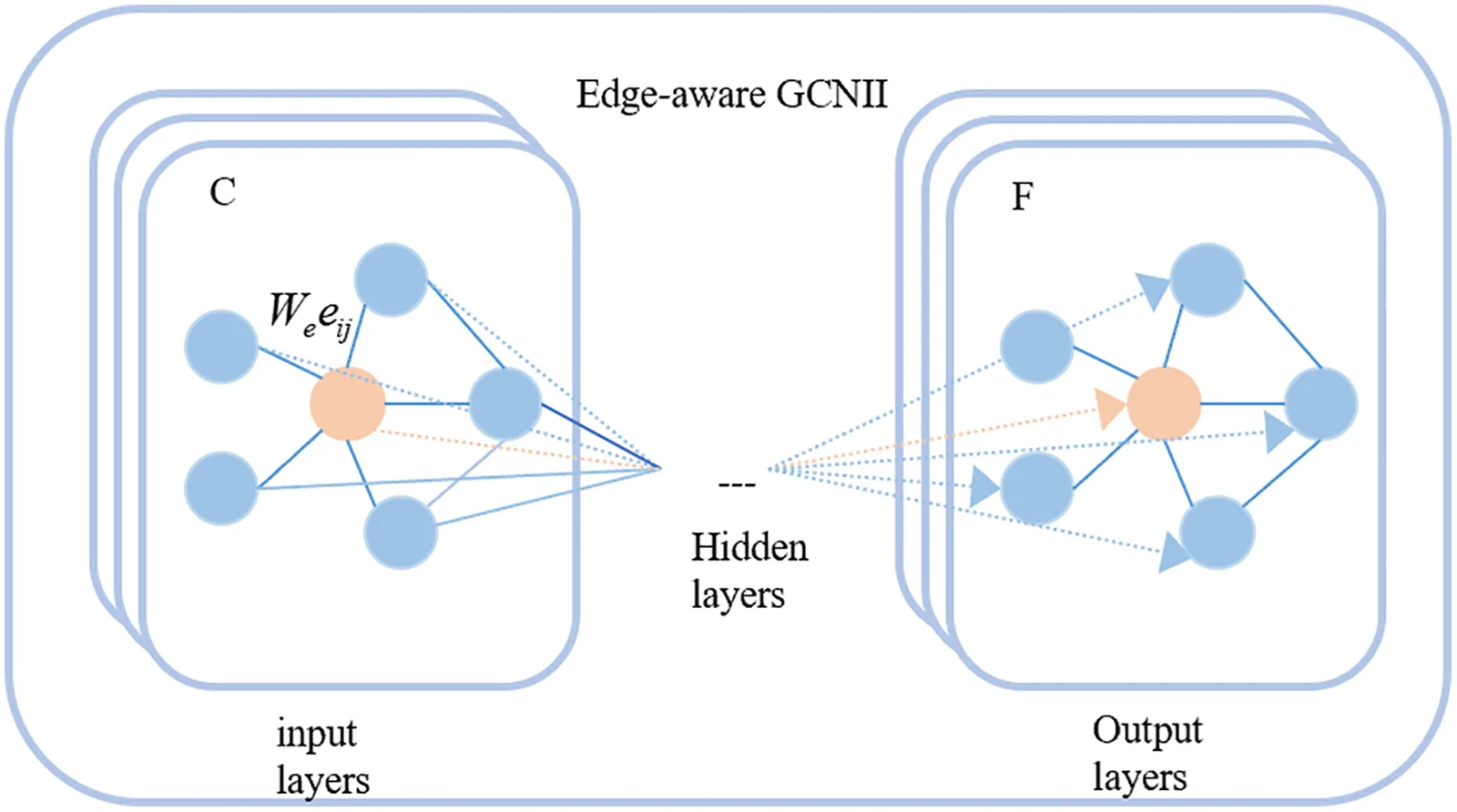
Edge-aware GCNII module.


$$\begin{array}{c} a_{ij}=w_{e}^{T}e_{ij},\;j\in N(i)\cup \left\{i\right\} \end{array}$$ (6)

Self-loop edges are assigned zero edge features as a neutral placeholder. Here, $w_{e}\in R^{d_{e}}$ denotes a learnable weight vector, and N(i) represents the set of neighboring nodes of node i. The attention coefficients $a_{ij}$, together with the edge weights $W_{ij}$, are utilized for neighborhood aggregation. To ensure the stability of information aggregation and incorporate self-connections, the adjacency matrix A is augmented with the identity matrix I and symmetrically normalized to obtain the edge-aware normalized adjacency matrix ${\tilde{A}}_{edge}$, as shown in Equations (7–9). Here, D denotes the degree matrix. The neighborhood aggregation representation of node i in the l−th layer is formulated as shown in Equation (10):


$$\begin{array}{c} \hat{A}=A+I \end{array}$$ (7)

$$\begin{array}{c} D_{ii}=\sum_{j}{\hat{A}}_{ij} \end{array}$$ (8)

$$\begin{array}{c} {\tilde{A}}_{edge}\left(i,j\right)=\frac{a_{ij}{\hat{A}}_{ij}}{\sqrt{D_{ii}D_{jj}}} \end{array}$$ (9)

$$\begin{array}{c} {\tilde{H}}_{i}^{\left(l\right)}=\sum_{j\in N(i)\cup \left\{i\right\}}{\tilde{A}}_{edge}\left(i,j\right)H_{j}^{\left(l\right)} \end{array}$$ (10)

The current-layer node features and the aggregated features are fused through the residual coefficient α to obtain the intermediate representation ${H^{\prime }}_{i}^{\left(l\right)}$, as shown in Equation (11). Subsequently, an identity mapping coefficient θ is applied to combine the intermediate representation with the initial features, yielding the output node features $H_{i}^{\left(l+\text{1}\right)}$ of the l−th layer, as shown in Equation (12):


$$\begin{array}{c} {H^{\prime }}_{i}^{\left(l\right)}=(\text{1}-\alpha )H_{i}^{\left(l\right)}+\alpha {\tilde{H}}_{i}^{\left(l\right)} \end{array}$$ (11)

$$\begin{array}{c} H_{i}^{\left(l+\text{1}\right)}=(\text{1}-\theta ){H^{\prime }}_{i}^{\left(l\right)}+\theta H_{i}^{\left(\text{0}\right)} \end{array}$$ (12)

Finally, the output is projected to the target dimension through a linear mapping, as shown in Equation (13):


$$\begin{array}{c} Y_{i}^{\left(l+\text{1}\right)}=W_{out}H_{i}^{\left(l+\text{1}\right)}+b \end{array}$$ (13)

Here, $W_{out}\in R^{d\times d^{\prime }}$denotes the learnable linear mapping matrix, $b\in R^{d^{\prime }}$ represents the bias vector, and $d^{\prime }$ is the target output dimension. Through the above mechanism, the Edge-Aware GCNII module can effectively alleviate the over-smoothing problem in deep networks while fully leveraging edge feature information to achieve differentiated neighborhood aggregation. This enables the model to preserve structural stability in deeper layers while assigning distinct weights to different types of chemical bonds (e.g., single, double, and aromatic bonds), thereby enhancing its sensitivity to local structural variations within molecular graphs.

In the MEGH-DDI framework, GAT is responsible for modeling local fine-grained interactions, dynamically computing neighbor node weights via self-attention to capture the heterogeneity and dynamic dependencies of shallow node representations; meanwhile, Edge-aware GCNII performs deep structural integration and chemical bond-aware modeling, alleviating over-smoothing through residual connections and identity mapping, and leveraging edge-feature attention to distinguish single, double, and aromatic bonds, thereby incorporating bond-level knowledge into node representations. The output of Edge-aware GCNII not only provides deep, smooth, and chemically enriched node features but also supports subsequent interpretability analyses, achieving a complementary synergy between the local dynamic perception of GAT and the deep stable encoding of Edge-aware GCNII.

### 3.4 Hop2Token and transformer

This study presents an innovative graph-structured Transformer architecture based on NAGphormer [29], with its core feature being the novel Hop2Token module. This module leverages graph diffusion techniques to transform the multi-hop neighborhood information of nodes into a serialized token representation. In the implementation process, feature propagation is first performed using the adjacency matrix to systematically capture the neighborhood features of each node across different hop distances. These features are then organized in order of increasing hop number to construct a complete sequential representation of the node. As detailed in Table 4, the Hop2Token module structurally integrates local and global graph information, providing a richer feature representation space for the graph Transformer model. This sequential processing approach not only preserves the topological properties of the graph but also enables a deep analysis of complex graph semantics through a learnable attention mechanism.

**Table 4:** Pseudocode of Hop2Token.

Algorithm 1 The Hop2Token Algorithm
Input: Normalized adjacency matrix ${\hat{A}}^{k}$; Feature matrix X; Propagation step K;
Output: Sequences of all nodes $X_{G}$;
1: for k=0 to K do
2: for i=0 to n-1 do
3: $X_{G}[i,k]=X[i]$;
4: end for
5: $X=\hat{A}X$;
6: end for
7: return Sequences of all nodes $X_{G}$;

The core functionality of this module lies in the intelligent transformation of graph-structured data into sequential information. It takes the updated node features ${\tilde{h}}_{i}^{\left(l\right)}={h^{\prime }}_{i}^{\left(l\right)}$ as input. Node features are iteratively aggregated using the adjacency matrix, with each aggregation representing an outward expansion of one hop. The K-hop neighborhood of each node is converted into a sequence, and the Transformer model is employed to capture the complex relationships among neighborhoods at different distance scales.

The 0-hop Token represents the initial features of a node ${\tilde{h}}_{i}^{\left(l\right)}$. The k-hop Token denotes the features of the k-th order neighbors, obtained through multiplication with the adjacency matrix $T_{k}^{\left(l\right)}=A^{k}{\tilde{h}}_{i}^{\left(l\right)}$ where k=1,2,..K. This process is iterated for max_hop (*K* times). Finally, the module stacks the aggregated features from all hops [0-hop, 1-hop,…, K-hop] to produce a multi-scale feature sequence for each node in the graph, as shown in Equation (14):


$$\begin{array}{c} X^{\left(l\right)}=\text{Stack}\left(\left[{\tilde{h}}_{i}^{\left(l\right)},A{\tilde{h}}_{i}^{\left(l\right)},A^{\text{2}}{\tilde{h}}_{i}^{\left(l\right)},\ldots ,A^{K}{\tilde{h}}_{i}^{\left(l\right)}\right]\right) \end{array}$$ (14)

This feature sequence is then fed into a Transformer encoder to model dependencies between different hops. The core of the Transformer comprises multi-head self-attention and position-wise feed-forward networks. This architecture is designed to capture global contextual information among different hop tokens in the sequence, thus producing more expressive context-enhanced representations. In the MHSA module, the self-attention mechanism enables the model to dynamically weigh the importance of other tokens when processing a particular token. To achieve this, the input sequence $S^{\left(l\right)}$ is first projected into three subspaces: query (Q), key (K), and value (V). The attention weights are then computed using scaled dot-product attention, formulated as shown in Equation (15):


$$\begin{array}{c} \text{Attention}\left(Q,K,V\right)=\text{softmax}\left(\frac{QK^{T}}{\sqrt{d_{k}}}\right)V \end{array}$$ (15)

Where, $Q=X^{\left(l-\text{1}\right)}W_{Q}$, $K=X^{\left(l-\text{1}\right)}W_{K}$, $V=X^{\left(l-\text{1}\right)}W_{V}$, $d_{k}=\frac{d}{n}um\_heads$. Under the multi-head mechanism, the features are split into multiple subspaces for parallel computation and then concatenated to form the output. This allows each token to perform weighted aggregation over all hop tokens based on the global context. The updated token representations are subsequently obtained through LayerNorm and MHSA, as expressed in Equation (16). Subsequently, the feed-forward network performs two successive linear transformations independently on each token, with a ReLU activation function applied to enhance the nonlinear representation capacity of the model, as shown in Equation (17):


$$\begin{array}{c} {\tilde{X}}^{\left(l\right)}=LayerNorm\left(X^{\left(l-\text{1}\right)}+MHSA\left(X^{\left(l-\text{1}\right)}\right)\right) \end{array}$$ (16)

$$\begin{array}{c} Z^{\left(l\right)}=LayerNorm\left({\tilde{X}}^{\left(l\right)}+FFN\left({\overset{\sim }{X}}^{\left(l\right)}\right)\right) \end{array}$$ (17)

To obtain the final vector representation of each node, the output sequence $Z^{\left(l\right)}$ from the Transformer encoder is passed through an attention pooling layer, which aggregates the tokens representing different hop neighborhoods into a single node embedding $h_{trans}^{\left(l\right)}$ enriched with multi-scale structural information, as shown in equations (18) and (19):


$$\begin{array}{c} \alpha_{k}=\frac{\;\;exp(w^{T}z_{k})}{\sum_{j=\text{0}}^{K}exp\left(w^{T}z_{j}\right)} \end{array}$$ (18)

$$\begin{array}{c} h_{trans}^{\left(l\right)}=\sum_{k=\text{0}}^{K}\alpha_{k}z_{k}^{\left(l\right)} \end{array}$$ (19)

Here, $w\in R^{d}$ is a trainable parameter, and the contribution of each token is determined by the attention weights $\alpha_{k}$.

### 3.5 Intra-graph and inter-graph feature extraction

To fully extract the rich structural features of drug graphs, this study employs a graph attention mechanism designed to capture both the internal structural dependencies within each drug and the complex interactions between different drugs [19]. Specifically, in terms of intra-view interactions, atoms within a drug graph are allowed to exchange attention information along their connected chemical bonds. Each atom aggregates the messages received from its neighboring atoms and updates its feature representation by integrating this information with its own characteristics.


$$\begin{array}{c} \alpha_{ij}=softmax\left(a^{T}\left[W_{h}|h_{i}^{\left(l\right)}|W_{h}h_{j}^{\left(l\right)}\right]\right) \end{array}$$ (20)

In Equation (20), $\alpha_{ij}$ denotes the attention weight between nodes i and j, while represent the feature vectors $h_{i}$ and $h_{j}$ of nodes i and j.$W_{h}$ and a are learnable parameters used to compute the attention scores.


$$\begin{array}{c} h_{i}^{\left(l+\text{1}\right)}=ELU\left(\sum_{j\in \left\{i\right\}\cup N_{i}}\frac{\text{1}}{c_{i}}\alpha_{ij}W^{\left(l\right)}h_{j}^{\left(l\right)}+b^{\left(l\right)}\right) \end{array}$$ (21)

In Equation (21), $h_{i}^{\left(l+\text{1}\right)}$ represents the node representation of the drug after the l+1 layer of the GAT. $N_{i}$ denotes the set of neighboring nodes of node i,$c_{i}$ is $\alpha_{ij}$ a normalization constant. The extraction of inter-graph structural features follows the core principles of intra-graph feature extraction.


$$\begin{array}{c} \beta_{ij}=softmax\left({\tilde{a}}^{T}\left[W_{k}|h_{i}^{\left(l\right)}|W_{k}h_{j}^{\left(l\right)}\right]\right) \end{array}$$ (22)

$$\begin{array}{c} {\tilde{n}}_{i}^{\left(l+\text{1}\right)}=ELU\left(\sum_{j\in \tilde{N}}\frac{\text{1}}{{\tilde{c}}_{i}}\beta_{ij}{\tilde{W}}^{\left(l\right)}n_{j}^{\left(l\right)}+{\tilde{b}}^{\left(l\right)}\right) \end{array}$$ (23)

As shown in Equations (22) and (23), the inter-graph adjacency matrix is represented as a bipartite graph $G:\left(d_{i},d_{j}\right)$ constructed from two drugs. In this graph G, $\tilde{N_{i}}$ denotes the neighbor set of node i, which corresponds to all atoms belonging to the other drug.

### 3.6 Adaptive Subgraph pooling structural information compression module

Compared with convolutional methods, current pooling techniques for graph data processing remain insufficient. Existing approaches generally exhibit notable limitations: traditional topological pooling focuses solely on structural information, while global pooling tends to overemphasize node features. Although hierarchical pooling methods such as DIFFPOOL [30] can construct hierarchical representations and support end-to-end graph coarsening, they suffer from a quadratic space complexity with respect to the number of nodes. To address this issue, this study employs SAGPooling [31], an attention-based adaptive subgraph pooling technique. This method breaks the conventional paradigm of neighborhood feature averaging by dynamically learning node importance, thereby enabling intelligent subgraph selection.

The core mechanism of this technique lies in an attention-based node scoring strategy: it first computes significance scores for each node based on the graph structure and node features. Subsequently, based on the node significance scores, the most important nodes are selected to construct a subgraph. Finally, the features of the selected nodes are aggregated to obtain the pooled subgraph representation. By adaptively selecting and aggregating subgraphs, SAGPooling can reduce the graph size while preserving essential information. This enhances the scalability and efficiency of the model and helps mitigate overfitting in graph neural networks. It can capture important local structures and relationships within the graph and provides an effective way to reduce graph size while enhancing the expressive capability of graph neural networks. The principle is illustrated in Equations (24) and (25):


$$\begin{array}{c} s^{\left(l\right)}=GNN_{SAG}\left(H^{\left(l\right)},A^{\left(l\right)}\right) \end{array}$$ (24)

$$\begin{array}{c} V^{\left(l+\text{1}\right)}=TopK\left(s^{\left(l\right)}\right) \end{array}$$ (25)

Here, $H^{\left(l\right)}$ denotes the node feature matrix at layer l, $A^{\left(l\right)}$ represents the corresponding adjacency matrix, $s^{\left(l\right)}$ denotes the node importance scores generated by the SAGPooling scoring function, and $V^{\left(l+\text{1}\right)}$ represents the set of nodes selected for the pooled subgraph.

### 3.7 Multimodal Feature Fusion and Classification Prediction Module

Node features are aggregated into graph-level representations through SAGPooling, while edge features are also integrated into the graph-level representation via linear transformation and pooling. Within each block, the final drug graph representation consists of three components, as expressed in Equation (26):


$$\begin{array}{c} h_{graph}=h_{gat}+h_{edge}+h_{gcn\_tran} \end{array}$$ (26)

Subsequently, as shown in Equation (27), this graph-level representation is fused with global molecular features (Mol2vec, MolT5) and normalized to obtain the final drug representation for the block. $h_{gat}$ originates from node pooling, $h_{edge}$ from edge feature pooling, and $h_{gcn\_tran}$ from the GCNII–Transformer fusion pathway.


$$\begin{array}{c} h^{\left(i\right)}=Norm\left(h_{graph}^{i}+h_{mol\text{2}vec}+h_{molT\text{5}}\right) \end{array}$$ (27)

The outputs of all Blocks are stacked into a three-dimensional tensor [B,L,D], where B represents the batch size, L represents the number of Blocks, and D represents the hidden dimension. This tensor is then fed into a Co-Attention module, which performs cross-graph attention interactions between the two drugs to generate the cross-drug representation. Finally, the model computes the interaction score by combining the representations of the two drugs through Co-Attention. For the DrugBank dataset, a relation-specific matrix $W_{r}$ is used to model drug–drug interactions. The scores computed for different block pairs are weighted by the Co-Attention coefficients $\alpha_{pq}$ and then summed to obtain the final score, as shown in Equation (28):


$$\begin{array}{c} s_{r}=\sum_{p=\text{1}}^{L}\sum_{q=\text{1}}^{L}\alpha_{pq}{\left(h_{p}^{\left(L\right)}\right)}^{T}W_{r}t_{q}^{\left(L\right)} \end{array}$$ (28)

For the ChChMiner dataset, which is a binary classification task without explicit relation types, the relation-specific matrix is omitted. Instead, the similarity scores between block-level representations of the two drugs are computed directly as ${\left(h_{p}^{\left(L\right)}\right)}^{T}t_{q}^{\left(L\right)}$, weighted by $\alpha_{pq}$, and summed to obtain the final interaction score.

To enhance the model’s ability in distinguishing positive and negative drug-drug interaction pairs while maintaining gradient stability during training, this study introduces an Adaptive Margin Sigmoid Loss (AMSL). This loss function extends the traditional Sigmoid loss by incorporating a temperature scaling factor, a margin offset, and a positive-negative sample weighting mechanism, thereby enhancing model generalization performance in complex drug-drug interaction tasks. To further stabilize training dynamics, an Adaptive Temperature Scaling mechanism is applied prior to loss computation to control the gradient distribution and the convergence rate of the model. Let the scores of positive samples be $p_{score}$ and those of negative samples be $n_{score}$. The temperature coefficient T is dynamically adjusted based on the standard deviation of the sample scores, as shown in Equation (29). The adaptive weights $w_{n}$ for negative samples are defined as in Equation (30).


$$\begin{array}{c} T=T_{\text{0}}\left(\text{1}+\lambda \cdot std\left(\left[p_{score},n_{score}\right]\right)\right) \end{array}$$ (29)

$$\begin{array}{c} w_{n}=softmax\left(\frac{n_{score}}{T}\right),n_{agg}=\sum_{n}w_{n}n_{score} \end{array}$$ (30)

The symbols represent the following:$T_{\text{0}}$ is the base temperature coefficient, λ is the adaptive scaling factor, and std(·) denotes the standard deviation of the sample scores. The loss terms for positive and negative samples are defined as follows, as shown in Equation (31):


$$\begin{array}{c} L_{pos}=-E\left[log\;\sigma \left(\frac{p_{score}-m}{T}\right)\right],\;L_{neg}=-E\left[w_{n}\cdot log\;\sigma \left(-\frac{n_{agg}+m}{T}\right)\right] \end{array}$$ (31)

In Equation (31), the margin offset m is used to enlarge the discrimination gap between positive and negative samples. The function E[·] denotes the average over the samples in a batch. The negative sample weight coefficients adjust the contribution of different samples to the loss, and the balancing coefficient β∈[0,1] is used to modulate the overall loss weight. The model loss function is defined as shown in Equation (32):


$$\begin{array}{c} L_{AMSL}=\beta L_{pos}+(\text{1}-\beta )L_{neg} \end{array}$$ (32)

Through the above mechanisms, the proposed loss function can maintain smooth gradients and prevent oscillations during the early stage of training, while in later stages, the margin offset m enlarges the separation between positive and negative samples, thereby improving model convergence speed and classification robustness. This loss function also effectively prevents gradient saturation and numerical instability, significantly enhancing the model's generalization ability in multi-modal feature fusion tasks.

## 4 Experimental results and analysis

### 4.1 Experimental setup and hyperparameters

To ensure reproducibility and clarity of the experimental setup, Table 5 summarizes the core hyperparameters used in model training. The optimizer, learning rate, batch size, number of epochs, weight decay, and early stopping patience are listed. Weight initialization follows Kaiming initialization for convolutional layers and Xavier initialization for linear layers. All model training experiments were conducted with a fixed random seed (42) to guarantee consistent results. In addition, negative sampling was performed with a ratio of 1:1 (neg_samples = 1), and the full dataset was used (data_size_ratio = 1.0).

**Table 5:** Model training hyperparameters.

Parameter category	Setting / Value
Optimizer	Adam
Learning Rate	1e-3
Weight Decay	1e-4
Batch Size	128
Number of Epochs	50
Early Stopping	Patience = 20
Loss Function	Adaptive Margin Sigmoid Loss
Weight Initialization	Kaiming for Conv, Xavier for Linear
Random Seed	42

### 4.2 Evaluation metrics

For the performance evaluation of the classification model, we adopted five core metrics: Accuracy (ACC), Area Under the Curve (AUC), Average Precision (AP), Recall, and the F1 Score. These metrics collectively provide a comprehensive assessment of the classification performance of the model from different perspectives. As a foundational metric, Accuracy intuitively reflects the degree of consistency between the model predictions and the true labels, with its specific calculation method detailed in Equation (33).


$$\begin{array}{c} ACC=\frac{TP+TN}{TP+TN+FP+FN} \end{array}$$ (33)

AUC, an important evaluation metric in machine learning, specifically refers to the area under the Receiver Operating Characteristic (ROC) curve. The magnitude of the AUC value directly reflects the discriminative ability of the classification model—when the AUC value approaches 1, it indicates that the model excels at distinguishing between positive and negative samples. The ROC curve is plotted in a Cartesian coordinate system, where the horizontal axis represents the False Positive Rate (FPR), defined as the probability of misclassifying negative samples, and the vertical axis denotes the True Positive Rate (TPR), indicating the proportion of correctly identified positive samples. Their calculation formulas are given in Equation (34) and Equation (35), respectively. It is important to emphasize that the AUC metric offers a distinct advantage for evaluating class-imbalanced datasets, as it comprehensively assesses the overall performance of the model across different decision thresholds.


$$\begin{array}{c} TPR=\frac{TP}{TP+FN} \end{array}$$ (34)

$$\begin{array}{c} FPR=\frac{FP}{TN+FP} \end{array}$$ (35)

Average Precision (AP) provides a comprehensive measure of model recognition performance and prediction ranking capability for specific categories. The calculation of this metric is based on an integrated analysis of the precision-recall curve: it first requires systematically adjusting classification thresholds to plot the precision versus recall curve, then obtains the final AP value by integrating the area under this curve. As shown in Equation (36), this computational process essentially quantifies the capability of the model to maintain high precision across different recall levels, effectively demonstrating the balanced performance of the detection algorithm between accuracy and coverage.


$$\begin{array}{c} AP=\sum_{n}\left(R_{n}-R_{n-\text{1}}\right)P_{n} \end{array}$$ (36)

Here, $P_{n}$and $R_{n}$ denote precision and recall at the n−th threshold. A higher AP value indicates better model capability to maintain an effective Precision-Recall trade-off across different thresholds.

Precision measures the proportion of true positives among all predicted positives, as mathematically defined in Equation (37). Recall, on the other hand, quantifies the ability of the model to identify actual positive samples, with the specific calculation given in Equation (38). These two metrics provide important quantified measures of model performance from two distinct perspectives: prediction accuracy and coverage completeness.


$$\begin{array}{c} Precision=\frac{TP}{TP+FP} \end{array}$$ (37)

$$\begin{array}{c} Recall=\frac{TP}{TP+FN} \end{array}$$ (38)

The F1 Score, a core metric for evaluating classification model performance, achieves an optimal balance between precision and recall by employing their harmonic mean. This characteristic makes it particularly suitable for datasets with highly imbalanced class distributions, effectively addressing the limitations of single-metric evaluation. As shown in Equation (39), its mathematical expression rigorously quantifies the trade-off between accuracy and completeness of the model.


$$\begin{array}{c} F\text{1}=\frac{\text{2}\times Precision\times Recall}{Precision+Recall} \end{array}$$ (39)

### 4.3 Evaluation of performance

#### 4.3.1 Baseline.

To validate the effectiveness of the proposed MEGH-DDI method in enhancing DDIs prediction performance, this study systematically compares it against multiple state-of-the-art baseline models, including SSI-DDI [15], GMPNN-CS [18], DGNN-DDI [21], DSN-DDI [19], MIRACLE [32], PEB-DDI [22]. The experimental design covers two key task scenarios: the transductive task, which evaluates prediction accuracy on known drug pairs, and the inductive task, which examines the generalization capability of the model to new drug pairs. Through this comprehensive evaluation, we demonstrate the applicability and reliability of MEGH-DDI in real-world drug combination prediction scenarios.

#### 4.3.2 Performance in transductive task.

The transductive task aims to evaluate the ability of the model to predict interactions among drugs that are already included in the training set. The experimental results of the transductive task on the DrugBank and ChChMiner datasets are presented in Tables 6 and 7. As shown, our model achieves the best performance on both datasets, consistently outperforming all comparative methods across all evaluation metrics.

**Table 6:** Experimental results of the DrugBank dataset in the transductive task.

Method	ACC(%)	AUC(%)	AP(%)	F1(%)
MR-GNN	96.04 ± 0.05	98.87 ± 0.04	98.57 ± 0.06	96.10 ± 0.05
MHCADDI	83.80 ± 0.27	91.16 ± 0.31	89.26 ± 0.37	85.06 ± 0.31
SSI-DDI	96.33 ± 0.09	98.95 ± 0.08	98.57 ± 0.14	96.38 ± 0.09
GAT-DDI	89.81 ± 1.00	95.21 ± 0.70	93.56 ± 0.90	90.18 ± 0.74
GMPNN-CS	95.30 ± 0.05	98.46 ± 0.01	97.94 ± 0.02	95.39 ± 0.05
SA-DDI	96.23 ± 0.01	98.80 ± 0.02	98.36 ± 0.04	96.29 ± 0.09
DSN-DDI	96.94 ± 0.02	99.47 ± 0.01	99.37 ± 0.02	96.93 ± 0.02
PEB-DDI	98.18 ± 0.04	99.65 ± 0.01	99.63 ± 0.03	98.19 ± 0.04
Ours	**98.36 ± 0.04**	**99.82 ± 0.03**	**99.79 ± 0.03**	**98.35 ± 0.03**

**Table 7:** Experimental results of the ChChMiner dataset in the transductive task.

Method	AUC(%)	AP(%)	F1(%)
GCN	82.84 ± 0.61	84.27 ± 0.66	70.54 ± 0.87
GIN	70.32 ± 0.87	72.41 ± 0.63	65.54 ± 0.97
GAT	85.84 ± 0.23	88.14 ± 0.25	76.51 ± 0.38
SEAL-CI	90.93 ± 0.19	89.38 ± 0.39	84.74 ± 0.48
NFP-GCN	92.12 ± 0.09	93.07 ± 0.69	85.41 ± 0.18
MIRACLE	96.15 ± 0.29	95.57 ± 0.19	92.26 ± 0.09
PEB-DDI	99.67 ± 0.12	99.95 ± 0.02	96.31 ± 0.55
Ours	**99.87 ± 0.04**	**99.98 ± 0.02**	**99.05 ± 0.08**

On the DrugBank dataset, DDI existence prediction remains challenging because the model needs to distinguish interacting drug pairs from non-interacting pairs across a diverse set of interaction mechanisms. Despite the diversity of the 86 interaction types represented in the original dataset, the prediction task is formulated as binary classification. Compared with existing methods, including MHCADDI and GAT-DDI, which achieve ACC values of 83.80% and 89.81%, respectively, MEGH-DDI achieves a substantially higher ACC of 98.36%, corresponding to improvements of 14.56 and 8.55 percentage points. These results demonstrate the strong capability of MEGH-DDI to discriminate between positive and negative drug pairs, despite the underlying diversity of DDI mechanisms.

On the ChChMiner dataset, since only binary classification is performed, the overall evaluation metrics are generally high. Nevertheless, our model still surpasses 99% across all three key metrics—AUC, AP, and F1. Specifically, the F1 score reaches 99.05%, representing an improvement of 2.74 percentage points over the current state-of-the-art model PEB-DDI (96.31%), and a remarkable increase of 28.51 percentage points compared with the baseline GCN model (70.54%). Although the ChChMiner dataset is only about 17% the size of DrugBank, our model consistently maintains high accuracy and strong robustness even under small-sample conditions.

In summary, the experimental results from the transductive task demonstrate the capability of the model to predict interactions among known drugs. By integrating dual-view representations of molecular fingerprints and molecular graphs, our model consistently maintains superior performance in binary classification scenarios. Moreover, it is able to learn effective molecular representations even on small-scale datasets, fully demonstrating its applicability and advantages in DDI prediction tasks.

#### 4.3.3 Performance in inductive task.

The inductive task aims to evaluate the ability of the model to predict interactions between drugs that were unseen during training, including interactions between new drugs or between a new and a known drug, which more effectively reflects the generalization capacity of the model. Overall, the model was evaluated on two partition settings across both datasets: S1 (new drug-new drug) and S2 (new drug-known drug). The experimental results of the inductive task on the DrugBank and ChChMiner datasets are presented in Tables 8 and 9. In contrast to the transductive task, the inductive setting requires the model to infer interactions for drugs or drug pairs not encountered during training, providing a more rigorous assessment of generalization. The results show that MEGH-DDI achieved the best performance on most evaluation metrics and remained competitive on the remaining metrics across the S1 and S2 settings.

**Table 8:** Experimental results of the DrugBank dataset in the inductive task.

Method	Inductive-S1				Inductive-S2			
	ACC(%)	AUC(%)	AP(%)	F1(%)	ACC(%)	AUC(%)	AP(%)	F1(%)
MR-GNN	62.63 ± 0.77	70.92 ± 0.84	73.01 ± 1.23	45.81 ± 2.51	74.67 ± 0.33	83.15 ± 0.60	83.81 ± 0.69	69.88 ± 0.86
MHCADDI	66.50 ± 0.62	72.53 ± 0.92	71.06 ± 1.61	67.21 ± 0.59	70.58 ± 0.94	77.84 ± 1.08	76.16 ± 1.45	72.74 ± 0.65
SSI-DDI	65.40 ± 1.30	73.43 ± 1.81	75.03 ± 1.42	54.12 ± 3.46	76.38 ± 0.92	84.23 ± 1.05	84.94 ± 0.76	73.54 ± 1.50
GAT-DDI	66.31 ± 0.61	72.75 ± 0.78	71.61 ± 1.00	68.68 ± 0.60	69.83 ± 1.41	77.29 ± 1.63	75.79 ± 1.95	73.01 ± 0.85
GMPNN-CS	68.57 ± 0.30	74.96 ± 0.40	75.44 ± 0.50	65.32 ± 0.23	77.72 ± 0.30	84.84 ± 0.15	84.87 ± 0.40	78.29 ± 0.16
SA-DDI	67.15 ± 0.88	73.62 ± 1.25	73.39 ± 1.40	63.40 ± 1.53	75.55 ± 1.12	82.95 ± 1.05	84.11 ± 0.92	71.94 ± 1.57
DSN-DDI	73.42 ± 1.29	81.79 ± 1.12	81.82 ± 1.48	70.34 ± 0.98	81.92 ± 1.20	91.01 ± 0.76	91.09 ± 0.93	80.18 ± 1.49
PEB-DDI	75.65 ± 2.34	83.08 ± 2.39	82.60 ± 3.26	**75.88 ± 2.12**	83.01 ± 0.70	91.94 ± 0.80	92.05 ± 1.01	81.52 ± 0.95
Ours	**76.48 ± 0.53**	**84.54 ± 1.15**	**84.93 ± 1.03**	74.48 ± 0.24	**83.66 ± 0.10**	**92.25 ± 0.54**	**91.79 ± 0.93**	**83.35 ± 0.51**

**Table 9:** Experimental results of the ChChMiner dataset in the inductive task (The average result of the three-fold calculation).

Method	Inductive-S1				Inductive-S2			
	ACC(%)	AUC(%)	AP(%)	F1(%)	ACC(%)	AUC(%)	AP(%)	F1(%)
GCN	70.63 ± 2.27	69.72 ± 0.54	79.14 ± 1.68	79.57 ± 0.04	73.49 ± 1.72	76.95 ± 3.49	84.97 ± 2.24	80.59 ± 0.23
GIN	69.30 ± 1.33	70.16 ± 0.16	81.91 ± 1.18	78.67 ± 1.37	74.60 ± 0.35	79.17 ± 1.19	86.03 ± 1.50	80.48 ± 0.23
SEAL-CI	68.91 ± 2.10	67.87 ± 3.05	77.68 ± 2.93	76.93 ± 1.62	69.34 ± 1.49	70.65 ± 2.48	79.60 ± 3.19	77.17 ± 1.48
NFP-GCN	72.53 ± 2.88	74.72 ± 3.66	83.32 ± 3.47	80.05 ± 2.76	81.60 ± 0.77	88.38 ± 0.81	92.80 ± 0.42	85.73 ± 0.53
MIRACLE	62.22 ± 2.99	55.54 ± 2.45	70.07 ± 3.84	72.09 ± 4.46	64.93 ± 1.68	69.07 ± 1.81	77.38 ± 1.53	71.69 ± 2.78
PEB-DDI	78.33 ± 2.19	82.03 ± 2.52	88.27 ± 2.97	84.76 ± 2.60	88.06 ± 1.46	93.60 ± 0.04	95.57 ± 0.53	91.08 ± 1.29
Ours	**84.01 ± 2.55**	**86.18 ± 3.06**	**92.63 ± 3.13**	**87.22 ± 3.24**	**90.50 ± 1.05**	**95.36 ± 1.12**	**97.02 ± 1.22**	**92.89 ± 0.35**

On the DrugBank dataset, under the Inductive-S1 setting, MEGH-DDI achieves the best performance in terms of ACC (76.48%), AUC (84.54%), and AP (84.93%), exceeding those of PEB-DDI by 0.83, 1.46, and 2.33 percentage points, respectively. However, its F1 score is 74.48%, which is slightly lower than that of PEB-DDI (75.88%). Overall, these results demonstrate that MEGH-DDI performs competitively under the Inductive-S1 setting, particularly in classification accuracy and ranking-based discrimination, although its F1 score remains slightly lower than that of PEB-DDI. Under the Inductive-S2 setting, MEGH-DDI achieves an ACC of 83.66%, an AUC of 92.25%, an AP of 91.79%, and an F1 score of 83.35%, corresponding to improvements of 0.65 and 1.83 percentage points in ACC and F1, respectively, compared with PEB-DDI. These results suggest that MEGH-DDI maintains competitive predictive performance when evaluated on drug pairs involving previously unseen drugs, supporting its ability to generalize beyond the drug pairs observed during training.

On the ChChMiner dataset, the model also achieves comprehensive superiority across both partition settings. Under the Inductive-S1 setting, it attains an ACC of 84.01%, an AUC of 86.18%, an AP of 92.63%, and an F1 score of 87.22%, representing improvements of 5.68%, 4.15%, 4.36%, and 2.46% over PEB-DDI, respectively, and significantly surpassing all baseline methods. Under the Inductive-S2 setting, the model further achieves an ACC of 90.50%, an AUC of 95.36%, an AP of 97.02%, and an F1 score of 92.89%. Compared with PEB-DDI, these results demonstrate additional gains of 2.44% in ACC, 1.76% in AUC, 1.45% in AP, and 1.81% in F1. These results indicate that the proposed model maintains exceptional predictive capability even in larger and sparser graph environments. In particular, substantial advantages in metrics such as F1 and AP, which reflect the balance between precision and recall, highlight the remarkable robustness and effectiveness of the model under small-sample and data-sparse conditions.

In summary, under the inductive task, the proposed model achieves competitive performance across the different partition settings on the two datasets, significantly surpassing existing methods. These results demonstrate that the proposed dual-view substructure representation approach is not only effective for predicting interactions among known drugs but can also be successfully generalized to scenarios involving new drugs or novel drug pairs, exhibiting strong generalization capability and substantial practical potential.

### 4.4 Ablation study

To systematically evaluate the contribution of each module and highlight the core innovations of the proposed MEGH-DDI framework, ablation experiments were conducted on the ChChMiner dataset under two settings (S1 and S2). As shown in Fig 4, the performance contributions of individual modules and their combinations are summarized, highlighting the complementary strengths of Hop2Token, Edge-aware GCNII, and global molecular features. A Graph Attention Network (GAT) was used as the baseline to capture local structural dependencies via self-attention on atomic neighborhoods, achieving ACC values of 77.44% (S1) and 85.32% (S2). While GAT effectively models immediate neighbor interactions, its shallow architecture and limited receptive field restrict its ability to capture long-range dependencies—a common limitation of standard GNNs in molecular modeling.

**Fig 4:**
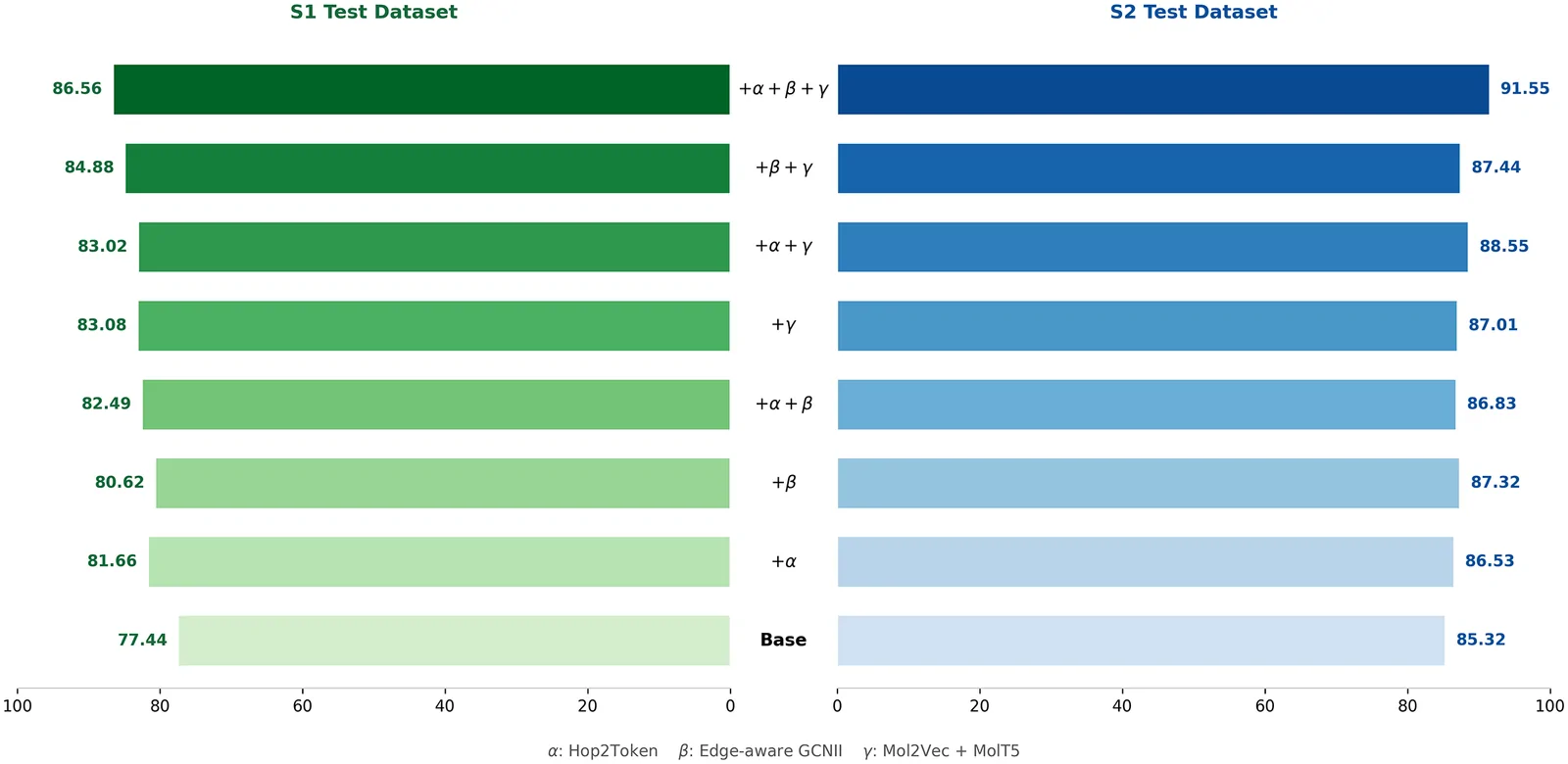
Comparison of module performance results (Using fold 2 as an example).

The Hop2Token module (α) was introduced to overcome this limitation. It serializes multi-hop neighborhoods (0-hop to K-hop) into token sequences and leverages a Transformer encoder to model dependencies across topological scales. Incorporating Hop2Token alone improved ACC to 81.66% (S1) and 86.53% (S2), representing gains of +4.22% and +1.21% over the baseline. This demonstrates that explicitly modeling higher-order neighborhood interactions enables the capture of long-range structural dependencies, such as long-range topological dependencies, which are critical for DDI prediction but often missed by shallow GNNs.

The Edge-aware GCNII module (β) addresses over-smoothing in deep GNNs while leveraging bond type information. Retaining GCNII residual connections and identity mapping allows deep propagation without performance degradation, and learnable edge-attention weights differentiate messages based on bond types (single, double, aromatic). When used individually, Edge-aware GCNII achieved ACC values of 80.62% (S1) and 87.32% (S2), outperforming the baseline by +3.18% and +2.00%. Improvements stem from the deeper 8-layer architecture and bond-aware message passing, which preserves chemical semantics compared to conventional GCNs that treat all neighbors uniformly.

Combining Hop2Token and Edge-aware GCNII (α+β) further improved ACC to 82.49% (S1) and 86.83% (S2), highlighting their complementary strengths: Hop2Token captures long-range topological patterns, while Edge-aware GCNII focuses on deep, bond-sensitive local propagation.

Global molecular features (γ), derived from Mol2vec and MolT5 embeddings, capture sequence-level semantics and functional group information often missed by graph-based modules. Individually, these features achieved ACC values of 83.08% (S1) and 87.01% (S2), surpassing the baseline and some graph-only combinations. Integrating these global features with Hop2Token (α+γ) or Edge-aware GCNII (β+γ) further increased ACC, demonstrating effective multi-modal fusion.

Integrating all three components into the full MEGH-DDI model yielded the best performance, with ACC values of 86.56% (S1) and 91.55% (S2), corresponding to improvements of +9.12% and +6.23% over the GAT baseline. These results validate that Hop2Token and Edge-aware GCNII synergize with global molecular features to provide a holistic drug representation, effectively integrating local bond-aware details, long-range topological patterns, and global chemical semantics, thereby achieving optimal predictive performance in DDI tasks.

As shown in Fig 5, the confusion matrices for the independent test sets S1 and S2 from the ChChMiner dataset indicate that the model exhibits strong classification capability across both test sets. On S1, the model correctly predicts a substantial number of positive samples (TP = 760) and negative samples (TN = 293), with misclassifications being comparatively low (FP = 91, FN = 91). Similarly, on S2, the model accurately distinguishes positive samples (TP = 6178) and negative samples (TN = 3281), with relatively few errors (FP = 313, FN = 601). These results demonstrate that the model effectively captures the interaction characteristics of drug pairs in both test scenarios, achieving high accuracy and reliability.

**Fig 5:**
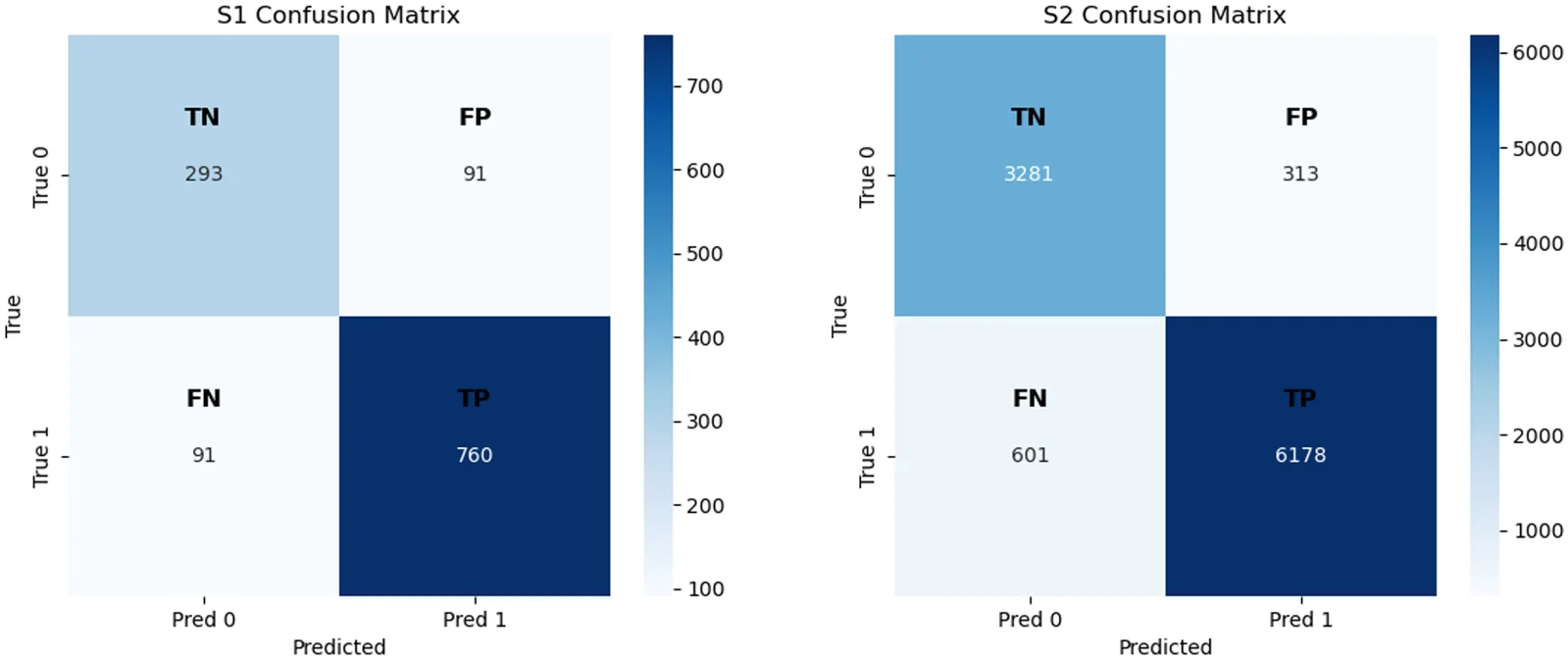
Confusion matrices of the independent test sets S1 and S2 on the ChChMiner dataset (Using fold 2 as an example).

## 5 Discussion

The experimental results highlight that MEGH-DDI generally outperforms existing baseline models across both transductive and inductive tasks, demonstrating its strong predictive power and generalization capability. The dual-view substructure representation, integrating Hop2Token, Edge-aware GCNII, and global molecular features, allows the model to effectively capture both local bond-level interactions and long-range topological dependencies, while also incorporating sequence-level chemical semantics. This multi-modal fusion is particularly advantageous for binary DDI prediction across datasets with diverse interaction patterns and varying data scales, where conventional GNNs may struggle due to limited receptive fields or over-smoothing. Furthermore, the substantial improvements observed in inductive settings indicate that MEGH-DDI can generalize to previously unseen drugs, suggesting its potential for real-world applications in drug discovery and safety assessment. Overall, these findings underscore the importance of jointly modeling local, global, and high-order structural information to achieve robust and accurate DDI prediction.

## 6 Interpretability analysis

To improve the transparency of MEGH-DDI, we analyzed model-derived attribution signals at the atom, bond, and multi-hop neighborhood levels. These signals indicate which molecular regions receive relatively high weights or have a stronger influence on model predictions.

### 6.1 Atom-level interpretability

To evaluate the capability of the model in identifying critical atoms for drug-drug interaction prediction, we calculated the contribution score for every atom in a given drug molecule. Specifically, for each atom, contribution values were obtained through pooling operations across different modules, and their average was taken as the overall score to quantify the importance of the atom within the entire network. Based on these scores, the most representative key substructures were extracted and visualized in Fig 6, highlighting atoms with higher contributions.

**Fig 6:**
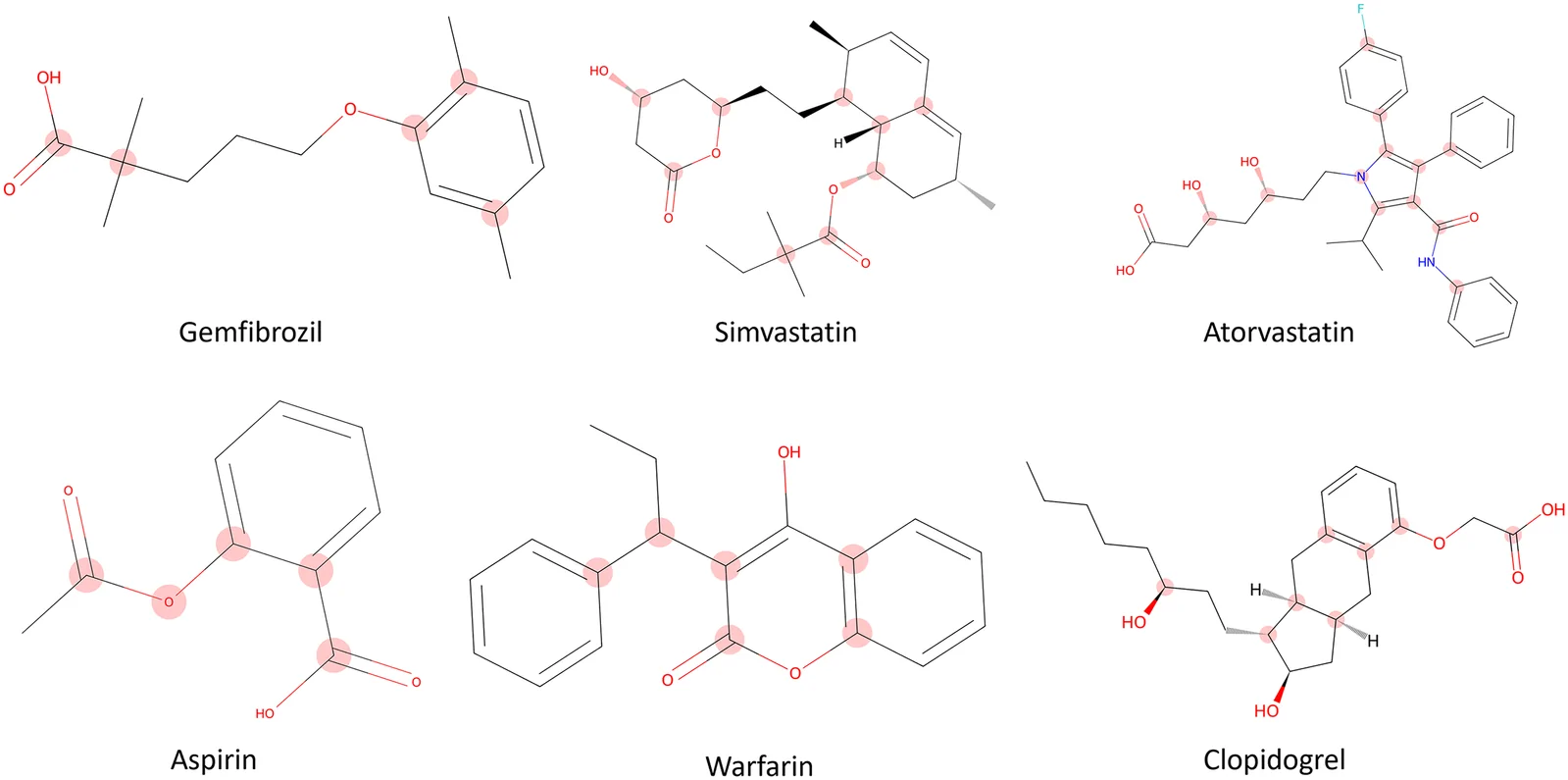
Key atom-level maps of drugs in DDI.

Table 10 presents five representative drug pairs used for attribution analysis. Co-administration of gemfibrozil and simvastatin can increase statin exposure and the risk of muscle-related toxicity. Aspirin combined with clopidogrel produces stronger antiplatelet effects but may increase bleeding risk, while clopidogrel combined with warfarin may further increase bleeding risk. Fluconazole may inhibit clozapine metabolism and increase its plasma concentration.

**Table 10:** Five representative DDI cases.

Drug name	Drug id	Drug name	Drug id
Gemfibrozil	DB01241	Simvastatin	DB00641
Atorvastatin	DB01076	Aspirin	DB00945
Aspirin	DB00945	Clopidogrel	DB00374
Clopidogrel	DB00374	Warfarin	DB00946
Fluconazole	DB01201	Clozapine	DB00214

To evaluate the influence of the highlighted atoms, we compared predicted DDI probabilities under three conditions: the complete molecular graph, masking of atoms with high attribution scores, and masking of randomly selected atoms. Masking highly attributed atoms produced a larger decrease in predicted probability than random masking in the examples shown in Fig 7, indicating that the model output was more sensitive to these atoms.

**Fig 7:**
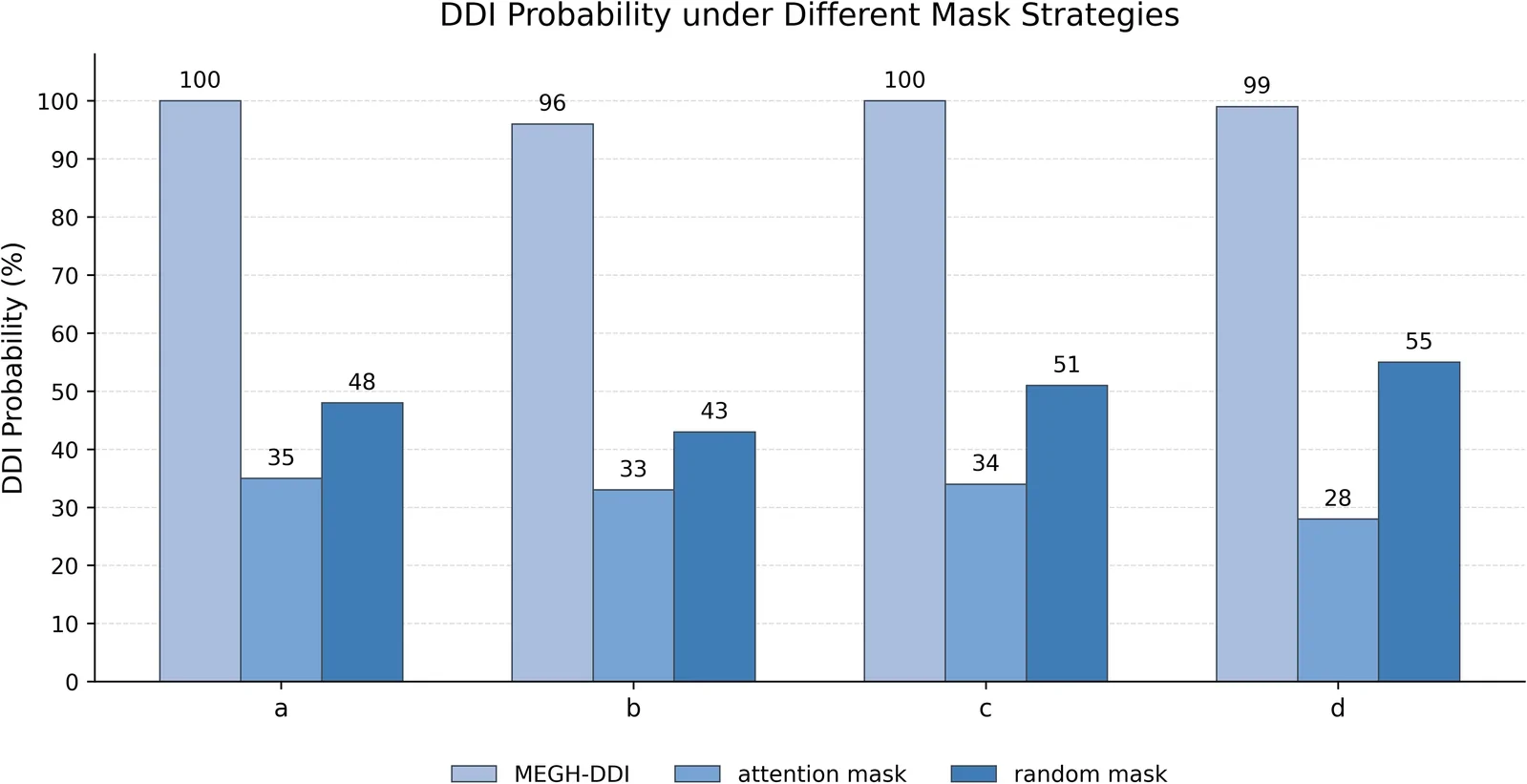
Effects of different masking strategies on DDI prediction probabilities.

### 6.2 Bond-level interpretability

We analyzed the edge weights generated by the Edge-aware GCNII module to identify chemical bonds that received relatively high model attention during DDI prediction. As shown in Fig 8, in the gemfibrozil-simvastatin example, high edge weights were observed around oxygen-containing regions of gemfibrozil and the lactone-containing region of simvastatin. In warfarin, the model emphasized bonds within or near the 4-hydroxycoumarin scaffold. For clopidogrel, high-weight regions included parts of the thiophene-containing structure.

**Fig 8:**
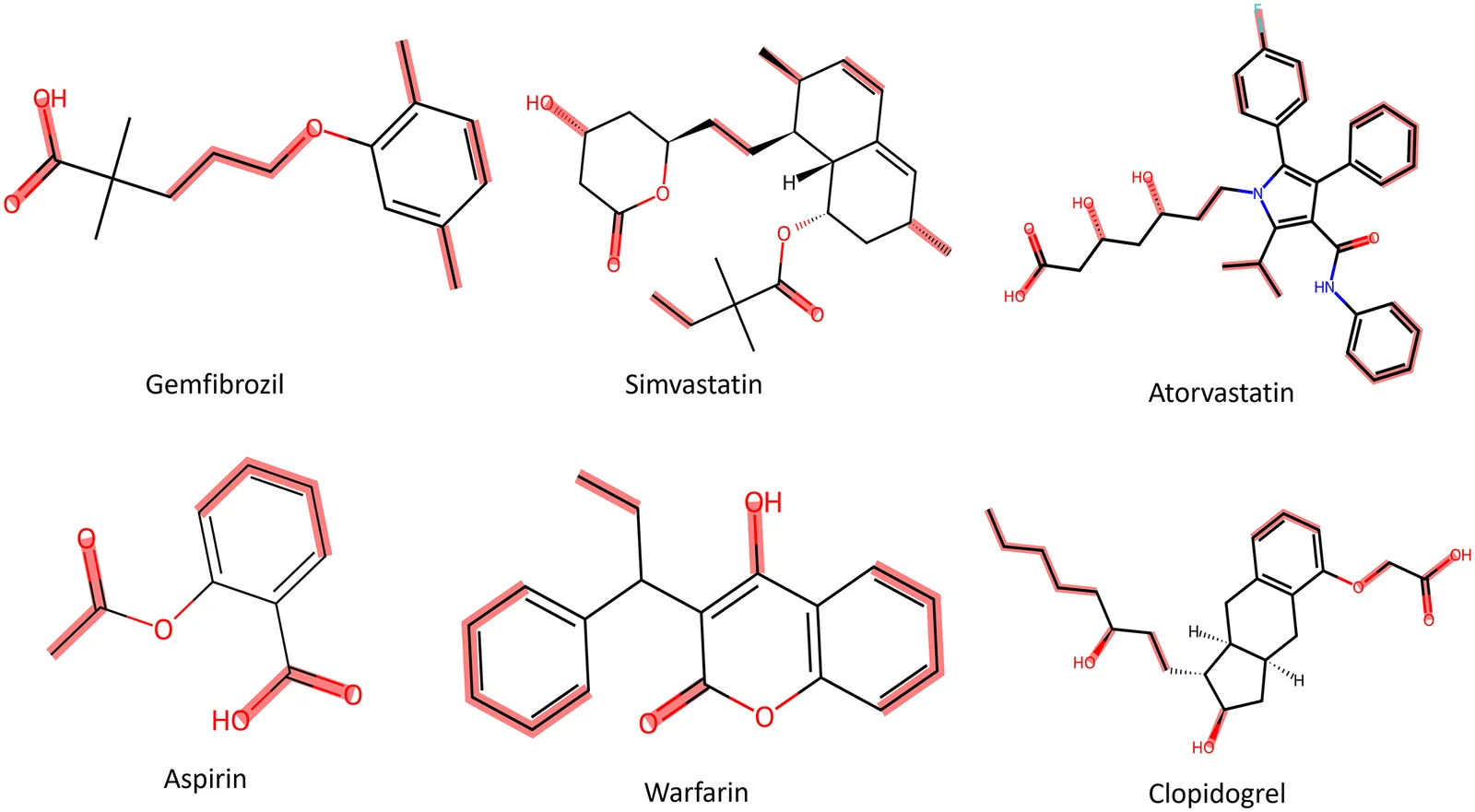
Visualization of attention on key chemical bonds in DDI.

These visualizations show which molecular regions contributed strongly to the model output and provide structure-level information for further investigation.

### 6.3 Mechanistic interpretability of DDI prediction based on multi-hop attention

The Hop2Token encoder transforms the multi-hop neighborhood of each atom (k = 0–3) into a token sequence. Here, 0-hop represents the atom itself, 1-hop represents atoms connected by one chemical bond, and higher-hop representations summarize broader neighborhoods in the molecular graph. The Transformer encoder assigns attention weights to the representations from different hop ranges.

As shown in Fig 9, for warfarin (DB00946), the attention weights were concentrated at k = 2 (0.280) and k = 3 (0.295). For clopidogrel (DB00374), the corresponding weights were 0.326 and 0.368. These results indicate that the model relies on information from broader graph neighborhoods rather than only the immediate neighborhoods of individual atoms.

**Fig 9:**
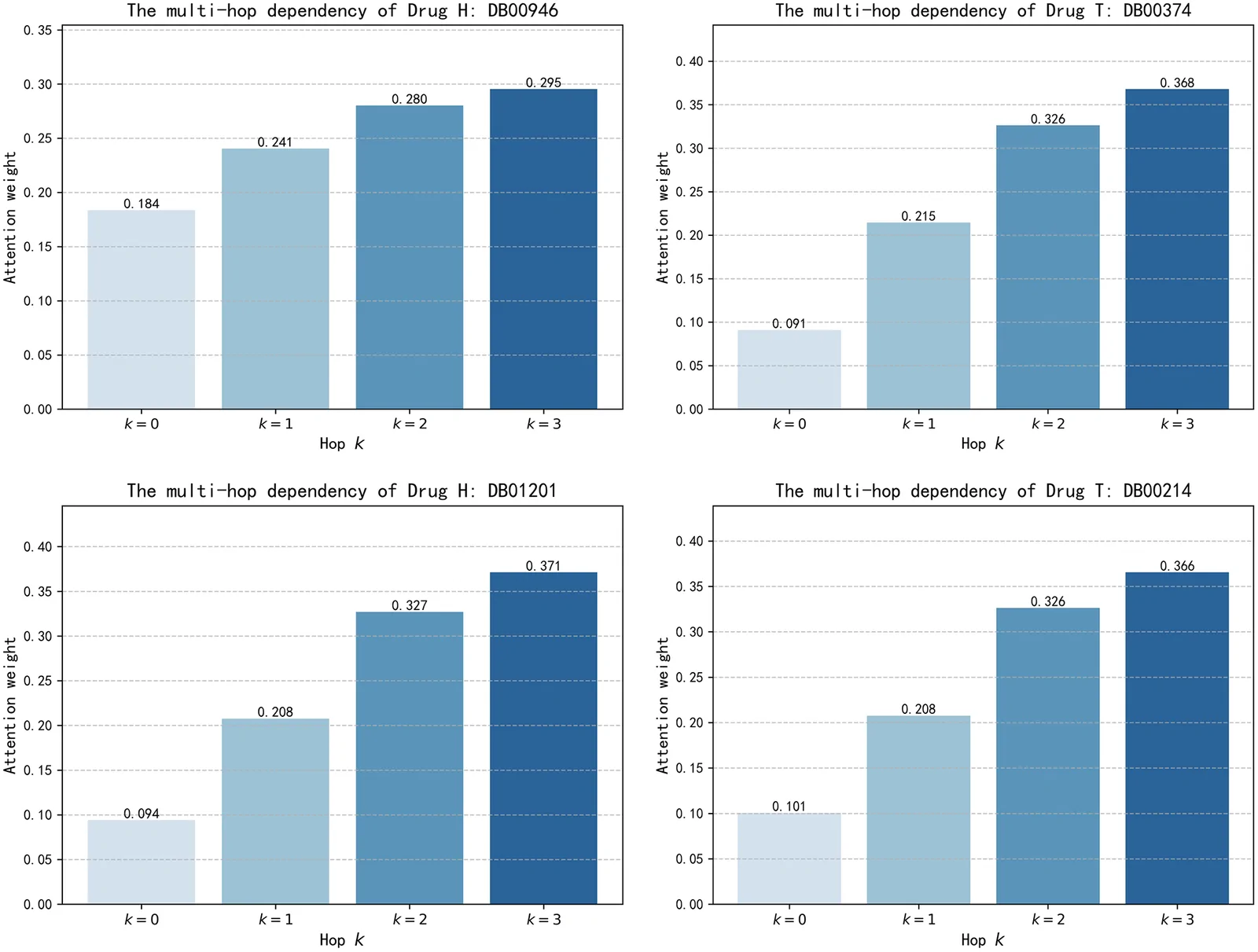
Attention-weight distributions across different hop ranges (k = 0-3) for representative drug molecules involved in DDI prediction.

## 7 Conclusion

In this study, we propose the MEGH-DDI framework, which achieves precise prediction of DDIs through multi-level molecular representations. Specifically, at the structural level, the model incorporates an edge-aware GCNII and employs residual and identity mappings to mitigate the over-smoothing problem inherent in deep GCNs while integrating a bond-type-based attention mechanism. This design enables the model to distinguish the differential influence of single, double, and aromatic bonds on the local environment, thereby enhancing atom-level node representations. For modeling higher-order dependencies, the Hop2Token multi-hop neighborhood encoding is employed to transform multi-hop node neighborhoods into sequential tokens, which are then processed by a Transformer encoder to capture cross-hop semantic interactions among substructures, enabling precise representation of key functional groups. At the molecular semantic level, cross-modal representations are integrated using Mol2vec and MolT5, with Mol2vec learning semantic vectors from large-scale molecular corpora based on substructure co-occurrence patterns, and MolT5 treating SMILES sequences as a chemical language to capture both syntactic and semantic features, thereby providing multi-scale, multi-modal global semantic information for nodes and molecules.

In large-scale experiments, MEGH-DDI achieved an accuracy of 98.36% and AUC/AP values exceeding 99.7% on the binary DDI prediction task using the DrugBank dataset, representing improvements of 14.56 and 8.55 percentage points in ACC over MHCADDI and GAT-DDI, respectively. On the binary classification task of ChChMiner, the model attained an F1 score of 99.05%, nearly 3 percentage points higher than PEB-DDI. Inductive evaluation demonstrated that the model maintains superior performance in predicting interactions involving unseen drugs and drug pairs, reflecting strong generalization capability and robustness in small-sample and complex drug spaces, thereby validating the effectiveness of edge-aware structural modeling and cross-modal semantic integration.

MEGH-DDI can be further extended to integrate three-dimensional molecular structures, quantum chemical properties, and clinical medication data, thereby establishing a more comprehensive multimodal framework for DDI prediction. Visualization of Hop2Token encoding and edge-level attention mechanisms enables the identification of key substructures driving specific interactions, providing an efficient, precise, and interpretable computational tool for drug safety assessment, polypharmacy optimization, and new drug development. The findings indicate that combining multi-scale structural information with cross-modal semantic representations constitutes an effective strategy for enhancing DDI prediction performance.

Moreover, MEGH-DDI may serve as a computational tool for the preliminary screening of candidate drug pairs. The model prediction scores can be used to prioritize drug combinations for further pharmacological and experimental evaluation.
