# Supplementary material for: MEGH-DDI: MolT5-driven edge-aware graph learning with Hop2Token representation for drug–drug interaction prediction
Source: PLoS Comput Biol. 2026 Oct 14;22(10):e1014393. doi: 10.1371/journal.pcbi.1014393 (PMC13693990; doi:10.1371/journal.pcbi.1014393)
Supplement: Supplementary file 1 [file pcbi.1014393.s002.docx]

**Revision Statement for Manuscript ID: PCOMPBIOL-D-25-02681**

"MEGH-DDI: MolT5-Driven Edge-Aware Graph Learning with Hop2Token Representation for Drug–Drug Interaction Prediction"

Dear Editors and Reviewers:

We are grateful to the Editor, and all the Referees for their valuable comments to make this work significantly improved. In this revised version, we have carefully addressed each and every question raised by all the Reviewers, and have tried our best to improve both quality and content of this paper. Please note that all the extensive changes made in the revised version are shown in yellow highlight.

Thanks in advance for reviewing the manuscript again.

Best regards,

Authors of the Manuscript ID: PCOMPBIOL-D-25-02681

**Response to the comments of referee #1**

**Comment 1**

Innovative Component Clarity: Although MEGH-DDI claims to fuse MolT5 with graph neural networks, the manuscript does not sufficiently articulate the unique contributions of the proposed Hop2Token encoding scheme or the Edge-aware GCNII design. As currently presented, the approach risks being perceived as a straightforward combination of existing techniques. The authors are encouraged to more clearly highlight the design motivations of these components and explain their fundamental differences from prior work.

**Response 1**

Thank you very much for your thorough review and insightful comments! We agree that the original manuscript did not sufficiently articulate the unique contributions of the Hop2Token encoding scheme and the Edge-aware GCNII design, and that the approach could be perceived as a straightforward combination of existing techniques. In response, we have thoroughly revised Section 3 to more clearly highlight the design motivations, the specific novelties of each component, and their fundamental differences from prior work.

The following is the corresponding section in the revised manuscript:

**Overview of model**

To comprehensively represent the multi-source features of drug molecules across chemical structures, topological relationships, and inter-graph interactions, this study proposes a multi-level graph learning framework that integrates graph neural network modeling with sequential Transformer-based inference. The framework introduces three key innovations designed to overcome limitations in existing DDI prediction methods: (1) an Edge-aware GCNII module that incorporates bond-type attention into deep graph convolutions to prevent over-smoothing while preserving chemical semantics; (2) a Hop2Token scheme that serializes multi-hop neighborhoods into token sequences, enabling Transformers to capture long-range dependencies across different topological scales; and (3) a multi-modal fusion strategy that combines graph-derived representations with global molecular embeddings (Mol2vec, MolT5) for holistic drug encoding. Through a modular design, the model progressively abstracts drug graphs from local atom-bond structures to global graph-level representations, ultimately enabling inter-drug interaction reasoning. The overall architecture consists of the following key components: first, a graph attention network (GAT) is employed to capture adaptive interaction weights between atoms via self-attention on node features, thereby enhancing the initial feature representations through weighted neighborhood aggregation. Subsequently, the Edge-aware GCNII convolution module balances topological smoothing and feature diversity during multi-layer propagation, with a specific emphasis on enhancing modeling capability for chemical bond information. To overcome the limitations of GNNs in modeling higher-order neighborhoods, Hop2Token and Transformer serialize multi-hop structural information into tokens, enabling explicit learning of the semantic contributions of different neighborhood ranges. At the graph-level stage, intra-graph and inter-graph attention mechanisms establish cross-feature interactions, and SAGPooling selects key substructures to form compact representations. Ultimately, multi-block graph representations are synergistically integrated with knowledge graph embeddings (RESCAL) to enable mechanistically interpretable DDI risk prediction.

**Comment 2**

Core Module Contributions: While the ablation studies indicate that each module contributes to performance improvements, the manuscript does not clearly distinguish which components constitute the core methodological innovations. The authors should explicitly identify the most critical innovations and, based on the ablation results, clarify which modules are primarily responsible for the observed performance gains and which limitations of existing DDI methods they address.

**Response 2**

Thank you for your insightful comment regarding the need to more clearly distinguish the core methodological innovations and their contributions. In response, we have substantially revised Section 4.3 (Ablation Study) to explicitly identify the two core innovations—Hop2Token and Edge-aware GCNII—and to clarify which limitations of existing DDI methods each module addresses.

Specifically:

1. Hop2Token (α) is identified as a core innovation that overcomes the limited receptive field of standard GNNs by serializing multi-hop neighborhoods into token sequences, enabling the Transformer to model long-range dependencies (e.g., allosteric effects) that are critical for DDI prediction but missed by shallow architectures. Ablation results show that adding Hop2Token alone improves ACC by +4.22% (S1) and +1.21% (S2) over the GAT baseline.
2. Edge-aware GCNII (β) is presented as the second core innovation, addressing two intertwined limitations: (a) the over-smoothing problem in deep GNNs, and (b) the underutilization of bond type information in message passing. By combining GCNII's deep propagation capabilities with learnable edge-attention weights, this module enables chemistry-aware aggregation. Alone, it achieves gains of +3.18% (S1) and +2.00% (S2).
3. Global molecular features (γ) are characterized as a complementary enhancement rather than a core innovation, as they capture sequence-level and functional group semantics that graph-based modules may miss. Their inclusion further boosts performance, but the primary methodological advances remain the Hop2Token and Edge-aware GCNII modules.

These revisions ensure that the core innovations are clearly distinguished, their contributions are quantitatively supported, and their relevance to overcoming limitations of prior work is explicitly articulated.

The following is the corresponding section in the revised manuscript:

**Ablation study**

To systematically evaluate the contribution of each module and highlight the core innovations of the proposed MEGH-DDI framework, ablation experiments were conducted on the ChChMiner dataset under two settings (S1 and S2). A Graph Attention Network (GAT) was used as the baseline to capture local structural dependencies via self-attention on atomic neighborhoods, achieving ACC values of 77.44% (S1) and 85.32% (S2). While GAT effectively models immediate neighbor interactions, its shallow architecture and limited receptive field restrict its ability to capture long-range dependencies—a common limitation of standard GNNs in molecular modeling.

The Hop2Token module (α) was introduced to overcome this limitation. It serializes multi-hop neighborhoods (0-hop to K-hop) into token sequences and leverages a Transformer encoder to model dependencies across topological scales. Incorporating Hop2Token alone improved ACC to 81.66% (S1) and 86.53% (S2), representing gains of +4.22% and +1.21% over the baseline. This demonstrates that explicitly modeling higher-order neighborhood interactions enables the capture of long-range structural dependencies, such as allosteric effects, which are critical for DDI prediction but often missed by shallow GNNs.

The Edge-aware GCNII module (β) addresses over-smoothing in deep GNNs while leveraging bond type information. Retaining GCNII residual and identity mapping allows deep propagation without performance degradation, and learnable edge-attention weights differentiate messages based on bond types (single, double, aromatic). When used individually, Edge-aware GCNII achieved ACC values of 80.62% (S1) and 87.32% (S2), outperforming the baseline by +3.18% and +2.00%. Improvements stem from the deeper 8-layer architecture and bond-aware message passing, which preserves chemical semantics compared to conventional GCNs that treat all neighbors uniformly.

Combining Hop2Token and Edge-aware GCNII (α + β) further improved ACC to 82.49% (S1) and 86.83% (S2), highlighting their complementary strengths: Hop2Token captures long-range topological patterns, while Edge-aware GCNII focuses on deep, bond-sensitive local propagation.

Global molecular features (γ), derived from Mol2Vec and MolT5 embeddings, capture sequence-level semantics and functional group information often missed by graph-based modules. Individually, these features achieved ACC values of 83.08% (S1) and 87.01% (S2), surpassing the baseline and some graph-only combinations. Integrating these global features with Hop2Token (α + γ) or Edge-aware GCNII (β + γ) further increased ACC, demonstrating effective multi-modal fusion.

Integrating all three components into the full MEGH-DDI model yielded the best performance, with ACC values of 86.56% (S1) and 91.55% (S2), corresponding to improvements of +9.12% and +6.23% over the GAT baseline. These results validate that Hop2Token and Edge-aware GCNII synergize with global molecular features to provide a holistic drug representation, effectively integrating local bond-aware details, long-range topological patterns, and global chemical semantics, thereby achieving optimal predictive performance in DDI tasks.

Fig 4. Comparison of module performance results.

**Comment 3**

Training Hyperparameter Details: Several important implementation details are either missing or scattered throughout the manuscript, including the choice of optimizer, learning rate, batch size, and number of training epochs, early stopping strategy, weight initialization scheme, and random seed settings. The authors are strongly encouraged to summarize all training hyperparameters in a unified table to improve clarity and reproducibility.

**Response 3**

Thank you very much for your thorough review and insightful comments! We agree that systematically organizing the key hyperparameters used during training helps improve the clarity of the methodological description and ensures reproducibility of the experimental results. In the revised manuscript, all implementation details have been thoroughly compiled, and a unified table of training hyperparameters has been added to the Methods section, providing a centralized overview of the main parameters used during model training. This table explicitly lists key settings such as optimizer type, learning rate, batch size, number of training epochs, and weight decay. Additionally, the revised manuscript further clarifies the early stopping strategy employed to prevent overfitting, the weight initialization method for network parameters, and the random seed settings used in the experiments, ensuring full reproducibility of the results.

**4.1 Experimental Setup and Hyperparameters**

To ensure reproducibility and clarity of the experimental setup, Table 5 summarizes the core hyperparameters used in model training. The optimizer, learning rate, batch size, number of epochs, weight decay, and early stopping patience are listed. Weight initialization follows Kaiming initialization for convolutional layers and Xavier initialization for linear layers. All experiments were conducted with a fixed random seed (42) to guarantee consistent results. In addition, negative sampling was performed with a ratio of 1:1 (neg_samples=1), and the full dataset was used (data_size_ratio=1.0).

Table 5. Model Training Hyperparameters.

| Parameter Category | Setting / Value |
| --- | --- |
| Optimizer | AdamW |
| Learning Rate | 1e-3 |
| Weight Decay | 1e-4 |
| Batch Size | 128 |
| Number of Epochs | 100 |
| Early Stopping | Patience=20 |
| Loss Function | BCEWithLogitsLoss |
| Weight Initialization | Kaiming for Conv，Xavier for Linear |
| Random Seed | 42 |

**Comment 4**

Clarifying GAT vs GCNII Roles: The manuscript employs both GAT and Edge-aware GCNII for graph feature modeling. Although both are neighborhood aggregation methods with weighting mechanisms, their specific roles and functional boundaries within the model remain unclear. The authors should further clarify the distinct modeling objectives of GAT and Edge-aware GCNII, discuss whether there is any functional overlap between them, and consider whether the model architecture could be simplified without compromising performance

**Response 4**

Thank you very much for your thorough review and insightful comments! In response, we have added clarifications on the functional roles of GAT and Edge-aware GCNII at the end of Section 3.3. Specifically, in the MEGH DDI framework, GAT is responsible for modeling local fine-grained interactions, dynamically computing neighbor node weights via self-attention to capture the heterogeneity and dynamic dependencies of shallow node representations. In contrast, Edge-aware GCNII performs deep structural integration and chemical bond-aware modeling, alleviating over-smoothing through residual connections and identity mapping, and leveraging edge-feature attention to distinguish single, double, and aromatic bonds, thereby incorporating bond-level knowledge into node representations. The output of Edge-aware GCNII not only provides deep, smooth, and chemically enriched node features but also supports subsequent interpretability analyses. This design enables a complementary synergy between the local dynamic perception of GAT and the deep stable encoding of Edge-aware GCNII, clarifying their distinct modeling objectives and functional boundaries within the model.

The following is the corresponding section in the revised manuscript:

**3.1 GAT**

GAT represents a breakthrough architecture in the field of graph deep learning, which innovatively integrates the attention mechanism with graph structural modeling. The model captures the varying influence of each node on its adjacent nodes by assigning different weights to neighboring nodes, thereby enabling precise representation and learning of graph-structured data. In GAT, the feature representation of each node is computed by aggregating the features of its neighboring nodes weighted by their corresponding attention scores. This mechanism enables the model to capture complex dependencies within the graph structure and effectively handle the heterogeneity and dynamics of graph data.

First, GAT is employed to update node features. Unlike standard GCNs that assign uniform or static weights to neighboring nodes, GAT dynamically computes attention weights for each neighbor through a self-attention mechanism. Let $h_{i}$ and$h_{j}$ denote the feature representations of nodes $i$ and $j$, let $W_{h}$ be the weight matrix for the linear transformation of node features. The attention coefficient between nodes$i$ and$j$ is computed as shown in Equation (1)：

$$e_{ij}=LeakyRelu(a^{T}[W_{h}h_{i}||W_{h}h_{j}])$$

Here,$a$denotes a learnable attentions vector,represents the concatenation operation,$LeakyRelu$is the activation function. The attention coefficients are normalized using the Softmax function to obtain the final attention weights$\alpha_{ij}$, as shown in Equation (2)：

$$\alpha_{ij}=\frac{\exp\left(e_{ij}\right)}{\sum_{k\in N\left(i\right)} \exp\left(e_{ik}\right)}$$

Where,$N_{\left(i\right)}$denotes the set of neighboring nodes of node$i$, Based on the computed attention weights, the features of neighboring nodes are aggregated to obtain the updated representation of node$i$, as shown in Equation (3)：

$$h_{i}^{'}=\sigma\left(\sum_{j\in N\left(i\right)} \alpha_{ij}Wh_{j}\right)$$

$\sigma\left(\cdot\right)$denotes the nonlinear activation function ReLU and$h_{i}^{'}$ represents the updated embedding of node $i$ at the current layer.

**3.2 GCN**

The Graph Convolutional Neural Network (GCN) has become a key technique in drug discovery tasks due to its exceptional capability in processing graph-structured data. Through its innovative graph convolution operation, the architecture effectively integrates topological relationships between nodes with their feature information, enabling multi-level semantic representation learning. Specifically, in the DDI prediction task, GCN first constructs a molecular graph based on atomic features and then performs intelligent aggregation of neighborhood features through a learnable adjacency matrix. The aggregated node embeddings are subsequently transformed using a nonlinear activation function to enhance the representational capacity of the model. Through this hierarchical and progressive information propagation mechanism, GCN gradually integrates local structural features with global topological information, ultimately producing node representations rich in semantic content. Its core computational process can be precisely described by the following mathematical expression (4):

$$H^{(l+1)}=\sigma(\tilde{D}^{-\frac{1}{2}}\tilde{A}\tilde{D}^{-\frac{1}{2}}H^{(l)}W^{(l)})$$

$\tilde{A}=A+I_{N}$represents the adjacency matrix *A* with added self-loops, $I_{N}$denotes the identity matrix, $\widetilde{D}^{-\frac{1}{2}}$is the inverse square root of the degree matrix used for normalization. This normalization ensures that each neighbor contributes proportionally during feature aggregation, preventing high-degree nodes from disproportionately influencing the aggregated representation. $W^{\left(l\right)}$is the weight matrix at the 𝑙-th layer for linear transformation of node features, and 𝜎 is the nonlinear activation function ReLU , which introduces nonlinearity to enable the model to capture complex patterns.

**3.3 Edge-Aware GCNII**

Notably, as network depth increases, GCNs encounter the challenge of representational homogenization — specifically, the over-smoothing phenomenon. This leads to a gradual loss of discriminative power in node features, thereby significantly constraining the potential for developing deeper models. To address this critical issue, the GCNII architecture (32) innovatively integrates a dual stabilization mechanism. Its initial residual connection technique preserves the original input features, thereby effectively maintaining feature diversity across network layers. Simultaneously, the introduced identity mapping operation significantly enhances the feasibility of training deep networks by stabilizing matrix transformations. Based on these two enhancements, the *ℓ-th* layer of GCNII can be formally expressed as shown in Equation (5)：

$$H^{(l+1)}=\sigma\left(\left(((1-\alpha_{l})\widetilde{P}H^{(l)}+\alpha_{l}H^{(0)}\right)\left((1-\beta_{l})I_{n}+\beta_{l}W^{(l)}\right)\right)$$

As shown in Equation (5), GCNII primarily combines the smoothed representation$\widetilde{P}H^{\left(l\right)}$ with the initial residual connection from the first layer$H^{\left(0\right)}$ and incorporates an identity$I_{n}$ mapping into the weight matrix $W^{\left(l\right)}$of the ℓ-th layer. This design effectively mitigates over-smoothing in deep networks while simultaneously enhancing the stability of parameter updates and overall trainability.

To address the importance of bond features in molecular graph modeling for message passing, this study proposes an Edge-Aware GCNII convolution module, as illustrated in **Fig 3**. This module preserves the two key mechanisms in GCNII for mitigating over-smoothing, namely the initial residual and identity mapping, while introducing learnable attention weights based on edge features. This allows the neighborhood aggregation process to distinguish between different types of edges. Specifically, the structural and chemical features of each edge in the graph are transformed through a linear mapping to obtain a scalar attention coefficient, which is then incorporated into the standard normalized neighborhood aggregation. The intra-layer coefficients α and θ are then combined to achieve the fusion of inter-layer residuals and initial features. Finally, a nonlinear transformation is applied to generate the node representations for the next layer. The initial node feature matrix is denoted as, where *N* represents the number of nodes and d denotes the dimensionality of node features. The input node representation of the *l-th* layer is denoted as. Each edge feature is represented as, where$d_{e}$ is the dimensionality of edge features, and the corresponding edge weight is denoted as$W_{ij}$, the residual coefficient is denoted as $\alpha\in\left[0,1\right]$, and the identity mapping coefficient is denoted as$\theta\in\left[0,1\right]$. The attention weight of each edge is computed by linearly mapping the edge feature into a scalar$a_{ij}$, as shown in Equation (6):

$$a_{ij}=w_{e}^{T}e_{ij},j\in N\left(i\right)\cup\left\{i\right\}$$

Here,denotes a learnable weight vector, and$N\left(i\right)$ represents the set of neighboring nodes of node *i*, including the self-loop node. The attention coefficients$a_{ij}$, together with the edge weights$W_{ij}$, are utilized for neighborhood aggregation. To ensure the stability of information aggregation and incorporate self-connections, the adjacency matrix *A* is augmented with the identity matrix *I* and symmetrically normalized to obtain the edge-aware normalized adjacency matrix$\widetilde{A}_{edge}$, as shown in Equations (7-9). Here, *D* denotes the degree matrix. The neighborhood aggregation representation of node *i* in the *l-th* layer is formulated as shown in Equation (10):

$$\widehat{A}=A+I$$

$$D_{ii}=\sum_{j} \widehat{A}_{ij}$$

$$\widetilde{A}_{edge}(i,j)=\frac{a_{ij}\widehat{A}_{ij}}{\sqrt{D_{ii}D_{jj}}}$$

$$\widetilde{H}_{i}^{(l)}=\sum_{j\in N\left(i\right)\cup\left\{i\right\}} \widetilde{A}_{edge}(i,j)H_{j}^{\left(l\right)}$$

The original node features and the aggregated features are fused through residual coefficient$\alpha$ to obtain the intermediate representation, as shown in Equation (11). Subsequently, an identity mapping coefficient$\theta$ is applied to combine the intermediate representation with the initial features, yielding the output node features$H_{i}^{\left(l+1\right)}$ of the *l-th* layer, as shown in Equation (12):

Finally, the output is projected to the target dimension through a linear mapping, as shown in Equation (13):

$$Y_{i}^{\left(l+1\right)}=W_{out}H_{i}^{\left(l+1\right)}+b$$

Here,denotes the learnable linear mapping matrix,represents the bias vector, and$d^{'}$ is the target output dimension. Through the above mechanism, the Edge-Aware GCNII module can effectively alleviate the over-smoothing problem in deep networks while fully leveraging edge feature information to achieve differentiated neighborhood aggregation. This enables the model to preserve structural stability in deeper layers while assigning distinct weights to different types of chemical bonds (e.g., single, double, and aromatic bonds), thereby enhancing its sensitivity to local structural variations within molecular graphs.

In the MEGH-DDI framework, GAT is responsible for modeling local fine-grained interactions, dynamically computing neighbor node weights via self-attention to capture the heterogeneity and dynamic dependencies of shallow node representations; meanwhile, Edge-aware GCNII performs deep structural integration and chemical bond-aware modeling, alleviating over-smoothing through residual connections and identity mapping, and leveraging edge-feature attention to distinguish single, double, and aromatic bonds, thereby incorporating bond-level knowledge into node representations. The output of Edge-aware GCNII not only provides deep, smooth, and chemically enriched node features but also supports subsequent interpretability analyses, achieving a complementary synergy between the local dynamic perception of GAT and the deep stable encoding of Edge-aware GCNII.

**Response to the comments of referee #2**

**Comment Major Issues**

The model integrates multiple heavy components, including Mol2Vec, MolT5, Edge-aware GCNII, and Hop2Token. Given the marginal performance gains, there is a clear risk of over-engineering. The authors are required to provide comprehensive ablation studies to demonstrate that the contributions of each module arise from meaningful structural modeling rather than parameter accumulation, and to discuss the feasibility of a simplified architecture.

**Respone Major Issues**

Thank you for the insightful comment regarding the potential risk of over-engineering in the proposed model. It is fully acknowledged that when multiple components are integrated, performance improvements must be justified by meaningful structural modeling rather than by parameter accumulation alone. In response to this concern, comprehensive ablation studies and additional analyses have been conducted to clarify the role and necessity of each module.

Section 4.3 has been expanded with systematic ablation experiments on the ChChMiner dataset. The results demonstrate that each module contributes consistent and non-trivial performance gains. Specifically, Hop2Token (α) improves accuracy by +4.22% on S1 and +1.21% on S2; Edge-aware GCNII (β) improves accuracy by +3.18% on S1 and +2.00% on S2; and global features (γ: Mol2Vec + MolT5) improve accuracy by +5.64% on S1 and +1.69% on S2. Notably, these gains are cumulative rather than additive. The full model (α + β + γ) achieves improvements of +9.12% on S1 and +6.23% on S2 over the GAT baseline, significantly outperforming all partial combinations. These results indicate that the modules are complementary rather than redundant, and that the observed improvements cannot be attributed to parameter growth alone.

Each component contributes through a distinct and principled modeling mechanism. The Hop2Token module explicitly models long-range topological dependencies by serializing multi-hop neighborhoods into token sequences, enabling attention-based reasoning across different structural scales that is not available in conventional GNN aggregation. The Edge-aware GCNII module introduces learnable edge-attention weights that differentiate message passing according to chemical bond types (e.g., single, double, and aromatic bonds), enabling chemically informed propagation beyond node-only GCN formulations. The global molecular features capture sequence-level semantics and functional group information that are difficult to infer solely from molecular graph topology. The complementary nature of these modules is further supported by the observation that all pairwise combinations outperform individual components, while the complete model consistently achieves the best performance.

The feasibility of a simplified architecture has also been carefully examined. Removing any single component leads to substantial performance degradation: excluding Hop2Token results in a 3.69% accuracy drop on S2; excluding Edge-aware GCNII leads to a 3.00% reduction; and excluding global features causes a 4.72% decrease. These results indicate that all three components are essential for optimal performance, and that a simplified architecture would compromise predictive accuracy, particularly under recall-sensitive and class-imbalanced settings. Although the model introduces additional complexity, the empirical gains justify the architectural design.

**Minor Issues**

**Comment 1**

  The abstract claims that MEGH-DDI improves performance by approximately 18 percentage points over the “previous best method,” whereas Table 1 shows that the improvement over the state-of-the-art baseline (PEB-DDI, 98.18%) is only 0.18%. This cross-scale discrepancy in performance reporting severely undermines academic credibility. The authors must clearly specify the reference baseline for the claimed 18% improvement. If this discrepancy results from a typographical error or selective comparison, it must be corrected accordingly.

**Response 1**

We sincerely thank you for keenly pointing out the apparent contradiction in the statement in the abstract—“an improvement of approximately 18 percentage points”—while the table shows only a 0.18% improvement over PEB-DDI. Thank you very much for your thorough review and insightful comments! We deeply apologize for this serious oversight and have carefully re-examined all the data in the manuscript. The situation is as follows: the statement in the abstract, “an improvement of approximately 18 percentage points over the existing state-of-the-art models,” was referring to the MHCADDI model (83.80%) and the GAT-DDI model (89.81%), not the PEB-DDI model (98.18%) listed in the table. The original wording indeed caused ambiguity, potentially misleading readers to believe that the improvement was relative to the strongest current baseline. In fact, MEGH-DDI (98.36%) achieves an improvement of 14.56 percentage points over MHCADDI (83.80%) and 8.55 percentage points over GAT-DDI (89.81%). The “approximately 18 percentage points” was a generalized summary of these two values without specifying the reference baseline, which led to the misunderstanding.

**The following is the corresponding section in the revised manuscript:**

**Abstract**

**…**

Experimental results demonstrate that MEGH-DDI achieves state-of-the-art performance across both transductive and inductive settings. On the DrugBank dataset, MEGH-DDI attains an accuracy of 98.36% in the transductive task, outperforming earlier baselines by a large margin and surpassing recent state-of-the-art methods, with both AUC and AP exceeding 99.7%. On the ChChMiner dataset, MEGH-DDI achieves an F1 score of 99.05%, improving upon the best existing method by 2.74 percentage points. Furthermore, in inductive tasks involving previously unseen drug pairs, MEGH-DDI consistently outperforms competing methods on both datasets under multiple inductive settings, demonstrating strong robustness and generalization capability in small-sample and complex DDI prediction scenarios.

**…**

**Comment 2**

The use of the SHAP framework for feature dimensionality reduction raises serious methodological concerns. SHAP is a post hoc explanation method based on model outputs. If feature selection is performed on the full dataset including the test set, it effectively introduces test-set information into the training process. The authors must explicitly clarify whether feature selection strictly adheres to training–test set isolation. Otherwise, the reported high accuracy may be invalidated due to data leakage.

**Response 2**

We sincerely thank you for pointing out the potential risk of test set information leakage during SHAP-based feature reduction. We fully acknowledge this concern and would like to clarify that in our original experimental design, feature selection strictly adhered to the train-test separation principle. The detailed procedure is as follows:

1. **Dataset Splitting:** For the DrugBank dataset, we adopted 3-fold cross-validation. For the ChChMiner dataset, we first split it into training, validation, and test sets in a 3:1:1 ratio. All subsequent operations were based on these fixed splits.
2. **Feature Selection Performed Only on the Training Set:** Within each cross-validation fold or fixed split, we trained a LightGBM classifier (for drug–drug interaction prediction) solely on the training set. Using the trained model, we computed SHAP values for each feature based on predictions on the training samples. By calculating the mean absolute SHAP value of each feature across all training samples, we obtained a feature importance ranking and finally selected the top 300 most important features.
3. **Application of Feature Selection Indices to All Sets:** After obtaining the top 300 feature indices, we applied the same indices to reduce the dimensionality of the MolT5 features (originally 1024-dimensions) in the training, validation, and test sets, keeping only the corresponding columns. The test set data were never used to train the LightGBM model, nor in computing SHAP values or feature importance rankings, fully preventing any data leakage.
4. **Independent Operations in Cross-Validation:** In DrugBank 3-fold cross-validation, the above procedure was independently repeated for each fold. Specifically, the training set of each fold was used to train a LightGBM model and compute SHAP values, select features, and then apply the selected feature subset to the corresponding validation and test sets. Therefore, the selected feature subsets may differ slightly across folds, but strict train-test separation was maintained in all cases.

It is important to emphasize that SHAP in this study was used solely as a supervised feature selection tool during the training stage, to identify the most discriminative feature subspace for the current task, rather than for model evaluation or interpretability analysis during testing. Methodologically, this SHAP-based process is entirely equivalent to other supervised feature selection strategies based on training data (e.g., feature importance derived from model weights or gradients) and does not introduce any prior knowledge from the test set.

We have added the above implementation details in Section 2.4 of the revised manuscript to further clarify the rigor of our feature selection procedure. We sincerely thank the reviewer again for the careful review, which has significantly improved the transparency and credibility of our methodological description.

The following is the corresponding section in the revised manuscript:

**2.4 Feature selection and dimensionality reduction based on SHAP**

Although the high-dimensional molecular representations generated by the MolT5 encoder (1024 dimensions in this study) contain rich information, they inevitably introduce computational redundancy and potential noise, which may negatively influence generalization performance of the model. To enhance the efficiency and stability of downstream prediction tasks while preserving the completeness of molecular representations, this study applies a feature selection strategy to intelligently compress high-dimensional vectors. Unlike traditional unsupervised dimensionality reduction techniques, such as PCA, which rely solely on data distribution, we innovatively employ the SHAP (SHapley Additive Explanations) framework to perform supervised feature selection, combining mathematical rigor with task-oriented guidance.

Importantly, the SHAP-based feature selection procedure does not introduce data leakage. The LightGBM model is trained exclusively on the training set, and SHAP values are computed only using training samples. Feature importance is determined based on the mean absolute SHAP values from the training data, and the resulting feature subset is fixed and directly applied to the validation and test sets without recomputation. At no stage are validation or test samples involved in feature selection, thereby ensuring a strictly leakage-free experimental protocol.

The specific implementation procedure consists of two main steps: First, a drug interaction prediction model is constructed using a LightGBM classifier. Subsequently, the SHAP explainer is applied to quantify the contribution weight of each feature to the prediction results. By computing the mean absolute value of the SHAP values for each feature and ranking them, we selected the top 300 key features from the initial 1024-dimensional set. This allowed us to construct a streamlined and effective drug representation vector. This process achieves not only dimensionality reduction from 1024 to 300 features but also ensures that the retained features are the most relevant for the drug interaction prediction task, thereby providing a more efficient and interpretable input space for subsequent modeling.

The experimental evaluation (as shown in **Table 3**) systematically compares the effects of various dimensionality reduction methods on drug-drug interaction prediction under the transductive setting. Although the original high-dimensional features demonstrate strong baseline performance (ACC=97.64%, AUC=99.67%), they exhibit clear computational efficiency bottlenecks. Although traditional unsupervised methods like PCA and UMAP improved overall metrics, their performance in sensitivity (SN) and F1-score remained unstable, indicating an insufficient retention of task-relevant information. While the ANOVA method preserved the discriminative power of features, it offered only limited performance improvement. In contrast, the SHAP-driven approach achieves breakthroughs across multiple metrics: accuracy increases to 98.90%, F1 reaches 99.38%, and sensitivity is notably improved to 99.03%, with feature importance being fully traceable. These results strongly demonstrate the unique advantage of the method in balancing model efficiency and predictive performance, providing the optimal feature reduction solution for this study.

…

**Comment 3**

In Section 5.3, the manuscript analogizes topological hop distance in chemical graphs to biological pathway steps. This analogy is scientifically untenable, as atomic-level topological distances within molecules and metabolic distances in macroscopic biochemical reaction networks represent fundamentally different concepts. The current explanation lacks biological grounding. The authors are requested to cite concrete pharmacological examples demonstrating that higher-weight hop values indeed correspond to interactions involving key functional groups.

**Response 3**

We sincerely thank you for pointing out the scientific issue in Section 5.3 regarding the analogy between topological hops and biological pathway steps. We fully acknowledge this criticism: atomic-level chemical graph topological distances and macroscopic biological pathway steps represent concepts at different scales, and the original analogy indeed lacked a biological basis. In the revised manuscript, we have removed this inappropriate analogy and re-explained the interpretability of multi-hop attention based on concrete pharmacological cases.

The hop number (k-hop) in the Hop2Token module is strictly defined within the chemical graph structure of the drug molecule: 0-hop represents the atom itself, 1-hop represents atoms directly connected via a single chemical bond, 2-hop represents atoms indirectly connected through two bonds, and so on. Therefore, higher hops (e.g., k = 2 or 3) merely indicate that the model considers more distant chemical neighborhoods during information aggregation, and do not directly correspond to metabolic steps or signaling cascades in biological pathways.

The following is the corresponding section in the revised manuscript:

**5.3 Mechanistic interpretability of DDI prediction based on multi-hop attention**

In this study, the MEGH-DDI framework employs the Hop2Token encoder to transform the multi-hop neighborhood of each node (k = 0–3) into a token sequence, and utilizes a Transformer encoder to generate attention weights for each hop. These attention weights quantify the model’s reliance on chemical structural information at different distances when predicting drug–drug interactions. It is important to clarify that the term k-hop is strictly defined within the chemical graph structure of the drug molecule: 0-hop represents the atom itself, 1-hop represents atoms directly connected via a single chemical bond, 2-hop represents atoms indirectly connected through two bonds, and so on. Therefore, higher-order attention weights merely indicate that the model considers more distant chemical neighborhoods during information aggregation, and do not directly correspond to metabolic steps or signaling cascades in biological pathways.

Nevertheless, by analyzing the distribution of attention weights across different hops, we can identify interactions that involve complex mechanisms requiring long-range structural information. For example, if a drug pair exhibits substantially higher attention weights for higher-order hops compared to lower-order hops, it may suggest that the interaction depends on functional groups or spatial conformations located far from the immediate reactive sites. Fig 9 illustrates the attention weight distributions across hops for four representative drugs. Taking the anticoagulant warfarin (DB00946) as an example, its attention is primarily concentrated at *k = 2* (0.280) and *k = 3* (0.295). Warfarin exerts its anticoagulant effect primarily through inhibition of VKORC1, and its coumarin scaffold constitutes the core pharmacophore associated with this activity. The elevated attention weights at higher hops indicate that the model tends to rely on multi-hop chemical topology spanning this scaffold, rather than focusing solely on local atomic neighborhoods. This observation suggests that the model aggregates long-range structural information to encode pharmacophore-related patterns, without implying a direct correspondence to specific biological binding steps.

For the prodrug clopidogrel (DB00374), the dependence on higher-order information is even more pronounced, with attention weights of 0.326 and 0.368 at *k = 2* and *k = 3*, respectively. This indicates that the model emphasizes extended structural regions involving the thiophene ring and ester linkage, which are known to be critical for its metabolic activation. To capture these structurally distributed functional groups and their connecting chains, the model must aggregate information across 2–3 hops within the chemical graph.

By leveraging multi-hop attention, the model is able to identify functional groups that are structurally distant from the central atom yet relevant to interaction-related patterns, thereby providing traceable chemical structure evidence for drug–drug interaction analysis. This chemistry-topology-based multi-hop attention analysis preserves scientific rigor while offering an intuitive and interpretable view of the model decision-making process.

**Comment 4**

Some of the web-based resources cited in the manuscript appear to be unstable or inaccessible. The authors should ensure that all referenced websites, online tools, or databases remain reliably accessible over time, or alternatively provide stable, permanent access links to ensure reproducibility and verifiability.

**Response 4**

Thank you very much for your thorough review and insightful comments! We fully agree that stable and accessible online resources are essential for ensuring the reproducibility and verifiability of research. We have thoroughly checked all web-based resources cited in the manuscript and updated the corresponding links where necessary. We also confirm that all referenced online tools and databases were accessible at the time of revision. For resources that may change over time, we have provided stable access links or official repository links whenever possible. These updates have been incorporated into the revised manuscript.

**Comment 5**

The manuscript uses PEB DDI as a baseline comparison, but this model is no longer considered state-of-the-art. The authors are encouraged to include comparative experiments with recently published advanced models (e.g., <https://doi.org/10.1016/j.compbiomed.2024.109496>) to comprehensively validate the performance, robustness, and effectiveness of the proposed approach.

**Response 5**

We sincerely thank you for the valuable suggestion of comparing our model with more recent advanced methods, such as the Jung and Yoo (2025) model. We fully acknowledge that including such comparisons could provide additional context for evaluating MEGH-DDI. However, a direct experimental comparison is not feasible due to differences in dataset settings. Specifically, Jung and Yoo (2025) employed the complete DrugBank dataset with 164 DDI types, whereas our experiments are conducted on the preprocessed 86-type subset provided by Shen et al. (2024). The substantial differences in data scale and class composition make one-to-one comparison of results on the full dataset inappropriate and could lead to misleading conclusions regarding relative performance.

To maintain fair and meaningful evaluation, we instead provide a detailed discussion of Jung and Yoo (2025) in the manuscript, summarizing their methodology and reported results, and contextualize our results under comparable benchmark settings used for SSI-DDI, DSN-DDI, and PEB-DDI. This ensures that readers can understand the state-of-the-art context without introducing invalid experimental comparisons.
