# Supplementary material for: MEGH-DDI: MolT5-driven edge-aware graph learning with Hop2Token representation for drug–drug interaction prediction
Source: PLoS Comput Biol. 2026 Oct 14;22(10):e1014393. doi: 10.1371/journal.pcbi.1014393 (PMC13693990; doi:10.1371/journal.pcbi.1014393)
Supplement: Peer review history

# Peer review: Decision Letter 0

**DOI:** 10.1371/journal.pcbi.1014393.r001

**Authors:** Dominik Wodarz, Amr Alhossary

2 Feb 2026

MEGH-DDI: MolT5-Driven Edge-Aware Graph Learning with Hop2Token Representation for Drug–Drug Interaction Prediction

PLOS Computational Biology

Dear Dr. Zuo,

Thank you for submitting your manuscript to PLOS Computational Biology. After careful consideration, we feel that it has merit but does not fully meet PLOS Computational Biology's publication criteria as it currently stands. Therefore, we invite you to submit a revised version of the manuscript that addresses the points raised during the review process.

Please submit your revised manuscript by Apr 04 2026 11:59PM. If you will need more time than this to complete your revisions, please reply to this message or contact the journal office at ploscompbiol@plos.org. When you're ready to submit your revision, log on to https://www.editorialmanager.com/pcompbiol/ and select the 'Submissions Needing Revision' folder to locate your manuscript file.

Please include the following items when submitting your revised manuscript:

* A letter that responds to each point raised by the editor and reviewer(s). You should upload this letter as a separate file labeled 'Response to Reviewers'. This file does not need to include responses to formatting updates and technical items listed in the 'Journal Requirements' section below.

* A marked-up copy of your manuscript that highlights changes made to the original version. You should upload this as a separate file labeled 'Revised Manuscript with Track Changes'.

* An unmarked version of your revised paper without tracked changes. You should upload this as a separate file labeled 'Manuscript'.

If you would like to make changes to your financial disclosure, competing interests statement, or data availability statement, please make these updates within the submission form at the time of resubmission. Guidelines for resubmitting your figure files are available below the reviewer comments at the end of this letter

We look forward to receiving your revised manuscript.

Kind regards,

Amr Ali Alhossary, PhD

Guest Editor

PLOS Computational Biology

Dominik Wodarz

Section Editor

PLOS Computational Biology

**Additional Editor Comments:**

One of the invited reviewers who accepted the invitation stopped responding. Therefore, in order to ensure a timely decision making, I am making the decision based on the other 2 reviewers. In case the remaining reviewer responds again, I will consider it and forward it to you.

**Journal Requirements:**

1) Please provide an Author Summary. This should appear in your manuscript between the Abstract (if applicable) and the Introduction, and should be 150-200 words long. The aim should be to make your findings accessible to a wide audience that includes both scientists and non-scientists. Sample summaries can be found on our website under Submission Guidelines:

https://journals.plos.org/ploscompbiol/s/submission-guidelines#loc-parts-of-a-submission

2) Please upload all main figures as separate Figure files in .tif or .eps format. For more information about how to convert and format your figure files please see our guidelines:

https://journals.plos.org/ploscompbiol/s/figures

3) Please amend your detailed Financial Disclosure statement. This is published with the article. It must therefore be completed in full sentences and contain the exact wording you wish to be published.

- State the initials, alongside each funding source, of each author to receive each grant. For example: "This work was supported by the National Institutes of Health (####### to AM; ###### to CJ) and the National Science Foundation (###### to AM)."

- State what role the funders took in the study. If the funders had no role in your study, please state: "The funders had no role in study design, data collection and analysis, decision to publish, or preparation of the manuscript.".

If you did not receive any funding for this study, please simply state: u201cThe authors received no specific funding for this work.u201d

**Reviewers' comments:**

Reviewer's Responses to Questions

**Comments to the Authors:**

**Please note here if the review is uploaded as an attachment.**

Reviewer #1: This paper proposes a multi-level graph learning framework, termed MEGH-DDI, for drug–drug interaction prediction. The proposed method integrates MolT5 and Mol2Vec representations with graph neural network architectures, and further incorporates GAT, Edge-aware GCNII, and a Hop2Token-based high-order substructure serialization mechanism to jointly model molecular structures and their high-order topological information. Experimental results on the DrugBank and ChChMiner datasets demonstrate that the proposed method outperforms existing approaches across multiple evaluation metrics, indicating promising predictive performance and application potential.To further improve the manuscript’s structure and enhance the persuasiveness of the findings, the following suggestions are provided:

1. Innovative Component Clarity: Although MEGH-DDI claims to fuse MolT5 with graph neural networks, the manuscript does not sufficiently articulate the unique contributions of the proposed Hop2Token encoding scheme or the Edge-aware GCNII design. As currently presented, the approach risks being perceived as a straightforward combination of existing techniques. The authors are encouraged to more clearly highlight the design motivations of these components and explain their fundamental differences from prior work.

2. Core Module Contributions: While the ablation studies indicate that each module contributes to performance improvements, the manuscript does not clearly distinguish which components constitute the core methodological innovations. The authors should explicitly identify the most critical innovations and, based on the ablation results, clarify which modules are primarily responsible for the observed performance gains and which limitations of existing DDI methods they address.

3. Training Hyperparameter Details: Several important implementation details are either missing or scattered throughout the manuscript, including the choice of optimizer, learning rate, batch size, and number of training epochs, early stopping strategy, weight initialization scheme, and random seed settings. The authors are strongly encouraged to summarize all training hyperparameters in a unified table to improve clarity and reproducibility.

4. Clarifying GAT vs GCNII Roles: The manuscript employs both GAT and Edge-aware GCNII for graph feature modeling. Although both are neighborhood aggregation methods with weighting mechanisms, their specific roles and functional boundaries within the model remain unclear. The authors should further clarify the distinct modeling objectives of GAT and Edge-aware GCNII, discuss whether there is any functional overlap between them, and consider whether the model architecture could be simplified without compromising performance

Reviewer #2: This manuscript presents a multimodal deep learning framework, termed MEGH-DDI, designed to predict drug–drug interactions (DDIs) by integrating SMILES-based semantic features (Mol2Vec, MolT5) with enhanced graph-structured representations (GAT, Edge-aware GCNII). The proposed approach introduces a Hop2Token module to capture higher-order topological dependencies of molecules and reports exceptionally high predictive performance on the DrugBank and ChChMiner datasets.

While the multimodal fusion strategy demonstrates a certain degree of technical complexity and novelty, the manuscript exhibits substantial deficiencies in data consistency, methodological rigor, and the logical coherence of biological interpretation, which require thorough clarification and revision by the authors.

Major problem:

The model integrates multiple heavy components, including Mol2Vec, MolT5, Edge-aware GCNII, and Hop2Token. Given the marginal performance gains, there is a clear risk of over-engineering. The authors are required to provide comprehensive ablation studies to demonstrate that the contributions of each module arise from meaningful structural modeling rather than parameter accumulation, and to discuss the feasibility of a simplified architecture.

Miner problems:

1. The abstract claims that MEGH-DDI improves performance by approximately 18 percentage points over the “previous best method,” whereas Table 1 shows that the improvement over the state-of-the-art baseline (PEB-DDI, 98.18%) is only 0.18%. This cross-scale discrepancy in performance reporting severely undermines academic credibility. The authors must clearly specify the reference baseline for the claimed 18% improvement. If this discrepancy results from a typographical error or selective comparison, it must be corrected accordingly.

2. The use of the SHAP framework for feature dimensionality reduction raises serious methodological concerns. SHAP is a post hoc explanation method based on model outputs. If feature selection is performed on the full dataset including the test set, it effectively introduces test-set information into the training process. The authors must explicitly clarify whether feature selection strictly adheres to training–test set isolation. Otherwise, the reported high accuracy may be invalidated due to data leakage.

3. In Section 5.3, the manuscript analogizes topological hop distance in chemical graphs to biological pathway steps. This analogy is scientifically untenable, as atomic-level topological distances within molecules and metabolic distances in macroscopic biochemical reaction networks represent fundamentally different concepts. The current explanation lacks biological grounding. The authors are requested to cite concrete pharmacological examples demonstrating that higher-weight hop values indeed correspond to interactions involving key functional groups.

4. Some of the web-based resources cited in the manuscript appear to be unstable or inaccessible. The authors should ensure that all referenced websites, online tools, or databases remain reliably accessible over time, or alternatively provide stable, permanent access links to ensure reproducibility and verifiability.

5. The manuscript uses PEB DDI as a baseline comparison, but this model is no longer considered state-of-the-art. The authors are encouraged to include comparative experiments with recently published advanced models (e.g., https://doi.org/10.1016/j.compbiomed.2024.109496) to comprehensively validate the performance, robustness, and effectiveness of the proposed approach.

**********

**Have the authors made all data and (if applicable) computational code underlying the findings in their manuscript fully available?**

The PLOS Data policy requires authors to make all data and code underlying the findings described in their manuscript fully available without restriction, with rare exception (please refer to the Data Availability Statement in the manuscript PDF file). The data and code should be provided as part of the manuscript or its supporting information, or deposited to a public repository. For example, in addition to summary statistics, the data points behind means, medians and variance measures should be available. If there are restrictions on publicly sharing data or code —e.g. participant privacy or use of data from a third party—those must be specified.

Reviewer #1: None

Reviewer #2: Yes

**********

PLOS authors have the option to publish the peer review history of their article (what does this mean?). If published, this will include your full peer review and any attached files.

If you choose “no”, your identity will remain anonymous but your review may still be made public.

**Do you want your identity to be public for this peer review?**  For information about this choice, including consent withdrawal, please see our Privacy Policy.

Reviewer #1: No

Reviewer #2: No

[NOTE: If reviewer comments were submitted as an attachment file, they will be attached to this email and accessible via the submission site. Please log into your account, locate the manuscript record, and check for the action link "View Attachments". If this link does not appear, there are no attachment files.]

**Figure resubmission:**

After uploading your figures to PLOS’s NAAS tool - https://ngplosjournals.pagemajik.ai/artanalysis, NAAS will process the files provided and display the results in the "Uploaded Files" section of the page as the processing is complete. If the uploaded figures meet our requirements (or NAAS is able to fix the files to meet our requirements), the figure will be marked as "fixed" above. If NAAS is unable to fix the files, a red "failed" label will appear above. When NAAS has confirmed that the figure files meet our requirements, please download the file via the download option, and include these NAAS processed figure files when submitting your revised manuscript.

**Reproducibility:**

?>

# Author comment: Author response to Decision Letter 1

**DOI:** 10.1371/journal.pcbi.1014393.r002

31 Mar 2026

Attachment Submitted filename: Response to the Reviewers.docx

# Peer review: Decision Letter 1

**DOI:** 10.1371/journal.pcbi.1014393.r003

**Authors:** Dominik Wodarz, Amr Alhossary

2 Jun 2026

Dear Ph.D. Zuo,

We are pleased to inform you that your manuscript 'MEGH-DDI: MolT5-Driven Edge-Aware Graph Learning with Hop2Token Representation for Drug–Drug Interaction Prediction' has been provisionally accepted for publication in PLOS Computational Biology.

Before your manuscript can be formally accepted you will need to complete some formatting changes, which you will receive in a follow up email. A member of our team will be in touch with a set of requests.

Please note that your manuscript will not be scheduled for publication until you have made the required changes, so a swift response is appreciated.

IMPORTANT: The editorial review process is now complete. PLOS will only permit corrections to spelling, formatting or significant scientific errors from this point onwards. Requests for major changes, or any which affect the scientific understanding of your work, will cause delays to the publication date of your manuscript.

Should you, your institution's press office or the journal office choose to press release your paper, you will automatically be opted out of early publication. We ask that you notify us now if you or your institution is planning to press release the article. All press must be co-ordinated with PLOS.

Thank you again for supporting Open Access publishing; we are looking forward to publishing your work in PLOS Computational Biology.

Best regards,

Amr Ali Alhossary, PhD

Guest Editor

PLOS Computational Biology

Dominik Wodarz

Section Editor

PLOS Computational Biology

***********************************************************

Reviewer's Responses to Questions

**Comments to the Authors:**

**Please note here if the review is uploaded as an attachment.**

Reviewer #1: The author has addressed my concern.

Reviewer #2: The authors addressed my comments

**********

**Have the authors made all data and (if applicable) computational code underlying the findings in their manuscript fully available?**

The PLOS Data policy requires authors to make all data and code underlying the findings described in their manuscript fully available without restriction, with rare exception (please refer to the Data Availability Statement in the manuscript PDF file). The data and code should be provided as part of the manuscript or its supporting information, or deposited to a public repository. For example, in addition to summary statistics, the data points behind means, medians and variance measures should be available. If there are restrictions on publicly sharing data or code —e.g. participant privacy or use of data from a third party—those must be specified.

Reviewer #1: None

Reviewer #2: None

**********

PLOS authors have the option to publish the peer review history of their article (what does this mean?). If published, this will include your full peer review and any attached files.

If you choose “no”, your identity will remain anonymous but your review may still be made public.

**Do you want your identity to be public for this peer review?**  For information about this choice, including consent withdrawal, please see our Privacy Policy.

Reviewer #1: No

Reviewer #2: No

# Editor report: Acceptance letter

**DOI:** 10.1371/journal.pcbi.1014393.r004

**Authors:** Dominik Wodarz, Amr Alhossary

PCOMPBIOL-D-25-02681R1

MEGH-DDI: MolT5-Driven Edge-Aware Graph Learning with Hop2Token Representation for Drug–Drug Interaction Prediction

Dear Dr Zuo,

I am pleased to inform you that your manuscript has been formally accepted for publication in PLOS Computational Biology. Your manuscript is now with our production department and you will be notified of the publication date in due course.

The corresponding author will soon be receiving a typeset proof for review, to ensure errors have not been introduced during production. Please review the PDF proof of your manuscript carefully, as this is the last chance to correct any errors. Please note that major changes, or those which affect the scientific understanding of the work, will likely cause delays to the publication date of your manuscript.

Soon after your final files are uploaded, unless you have opted out, the early version of your manuscript will be published online. The date of the early version will be your article's publication date. The final article will be published to the same URL, and all versions of the paper will be accessible to readers.

For Research, Software, and Methods articles, you will receive an invoice from PLOS for your publication fee after your manuscript has reached the completed accept phase. If you receive an email requesting payment before acceptance or for any other service, this may be a phishing scheme. Learn how to identify phishing emails and protect your accounts at https://explore.plos.org/phishing.

Thank you again for supporting PLOS Computational Biology and open-access publishing. We are looking forward to publishing your work!

With kind regards,

Sharmila Kamatchi

PLOS Computational Biology | Carlyle House, Carlyle Road, Cambridge CB4 3DN | United Kingdom ploscompbiol@plos.org | Phone +44 (0) 1223-442824 | ploscompbiol.org | @PLOSCompBiol
